# Synthesis, characterization, computational and dyeing behavior of Cu(II) and Zn(II) metal complexes derived from azo-Schiff bases containing phenol derivatives

**DOI:** 10.1186/s13065-025-01571-6

**Published:** 2025-07-10

**Authors:** Hemmat A. Elbadawy, Ali Eldissouky, Morsy Ahmed El-Apasery, Doaa S. Elsayed, Entesar. A. Alaswad

**Affiliations:** 1 Chemistry Department, Faculty of Science, Alexandria University, Alexandria, Egypt; 2 Dyeing, Printing and Textile Auxiliaries Department, Textile Research and Technology Institute, National Research Centre, Giza, Egypt; 3 Chemistry Department, Faculty of Science, Sebha University, Sebha, Libya

**Keywords:** Azo-schiff bases, Sulfanilamide, Biological activity, DFT, Transition metal complexes, Molecular docking, Dyeing properties

## Abstract

This study discusses the synthesis, characterization, and applications of two novel azo-Schiff bases, HL^1^ and HL^2^, derived from HL, 4-((3-formyl-4-hydroxyphenyl)diazenyl)benzenesulfonamide with *o*-aminophenol and *o*-amino-thiophenol, respectively, and their Cu(II) and Zn(II) complexes. Stability, dyeing, biological activity, and computational investigations with experimental data are examined. HL^1^, HL^2^ and their metal complexes were fully characterized utilizing different physicochemical techniques, such as UV-Vis, FTIR, mass spectra, thermal analysis (TGA, DTG and DTA), as well as EPR, molar conductivity measurements for metal complexes. The experimental findings showed that the metals were ligated to the azo-Schiff bases, HL^1^ and HL^2^ via the azomethine-N and phenolic-O or thiolato-S, respectively. The properties of the compounds as polyester fabric dyes were examined. They were evaluated for color fastness to washing, rubbing, and perspiration using ISO 105-C02:1989, X12:1987, and E04:1989. The colored materials’ superior intra-fiber diffusion inside the substrate may explain their exceptional washing and rubbing and perspiration fastness. In vitro antimicrobial efficiency of all products was explored against a panel of pathogenic bacteria, including Gram-positive bacteria *Staphylococcus aureus* and *Bacillus subtilis*, Gram-negative bacteria *Escherichia coli* and *Proteus vulgaris*, and pathogenic fungi *Aspergillus flavus* and *Candida albicans*, compared to Gentamycin and Ketoconazole as standards. Additionally, the cytotoxicity of developed compounds in one human cell line, lung carcinoma (A-549), was examined, demonstrating how chelation and molecular structure can affect anticancer activity. Compared to Ascorbic acid, their antioxidant activities were evaluated, and their ability to reduce DPPH radical was affected by molecular composition and structure. For free ligands, HL^1^ > HL > HL^2^ decreased power. For metal complexes, [Cu(L^1^)₂]0.5 H₂O was more potent than [Zn(L^1^)₂].2H₂O, but [Zn(L^2^)₂].2H₂O >[Cu(L^2^)₂]0.3 H₂O, as antioxidant. The applied computational studies and molecular docking on the proposed structures of the complexes were in good agreement with the experimental results.

**Supplementary Information:**

The online version contains supplementary material available at 10.1186/s13065-025-01571-6.

## Introduction

Azo Schiff bases have both azomethine and azo groups, which exhibit strong donor and important characteristics in coordination chemistry. Schiff bases and azo compounds are recognized for their antifungal, antibacterial, dyeing, and herbicidal properties [1–3]. The azomethine group’s exceptional ability to transfer electrons allows it to form stable complexes with various transition metal ions [4–8]. Despite both groups possessing desirable donor features, it has been found that coordination of the azomethine group is more advantageous [9, 10]. Azo compounds make up a significant portion of the synthetic dye industry and are considered the largest family of dyes globally [4, 11–13]. In addition, heterocyclic Azo-dyes function as markers for anatomical research, pathologic staining, and applicable in common analytical processes. They are representing an important category of dyeing materials [14]. The previous two decades have focused on synthesizing Azo-Schiff bases, coordinating them with different transition metals, and evaluating their antimicrobial and pharmaceutical properties for human benefit. On the other hand, sulfanilamide (SN), sometimes referred to as 4-aminobenzene sulfonamide, has an active functional group -S(= O)_2_–NH_2_, which is the consequence of substituting the amino group in sulfonic acid for the hydroxyl group. The sulfonyl group plays very important role as a major component of the number of biologically active molecules [15]. The sulfonamide-based Schiff bases are studied for their antioxidant, anticancer, and antimicrobial activities, showing promising results [16–18]. In addition, Schiff bases and Salicylaldehyde-derived Schiff bases are recognized as polydentate ligands that can coordinate with metal ions in both their deprotonated and neutral states [2, 19–21]. The metal complexation sometimes provides extra biological activity to Schiff bases, as well as anticancer activity [8, 14, 22]. Not only Schiff bases and their metal complexes, but also many chemically synthesized azo dyes and sulfonamide drugs were recognized as early antimicrobial agent [23–25]. Recently, metal-based pharmaceuticals are employed in the treatment of several ailments, including cancer, and inflammatory and cardiovascular disorders [26–28]. Starting out, cisplatin-based chemotherapy was widely utilized; however, due to its limitations, researchers began to design and synthesize novel non-platinum-based complexes for therapeutic applications. Consequently, the design and analysis of novel transition metal and lanthanide complexes with various ligands, such as azo dyes, Schiff bases, and azo-Schiff bases, have been pursued [29, 30]. Among several Metal ions, Cu(II) and Zn(II) complexes play important roles in drug design investigations [31].

Furthermore, the nuclear and electronic structures of molecules in their lowest energy state are extensively analyzed in chemistry and materials science by density-functional theory (DFT) [32–34]. Density Functional Theory (DFT) pertains to functionals associated with spatially varying electron density, covering structural properties (bond angles and lengths), reactivity descriptors, frontier molecular orbitals (FMO), Mulliken atomic charges (MAC), molecular electrostatic potential (MEP), and electronic characteristics. It provides insights into multi-electron systems and is extensively recognized across diverse disciplines, particularly in computational chemistry, due to its versatility and broad applicability [35, 36]. Integrating computational analyses with experimental findings enhances comprehension of material characteristics, potentially resulting in the development of novel uses [37]. Additionally, one low-cost theoretical approach to modeling the interaction between possible medications and cell receptors is molecular docking (MD). Importantly, this strategy can be used to choose the most promising medicine before research experiments [32, 38–42].

Based on the importance of the aforementioned materials and in pursuit of multiple applications for these compounds, specifically dyeing and biological activity, the aim of this study is to develop and evaluate novel azo-Schiff bases derived from the amino-sulfonamide group, along with their corresponding metal complexes. Physicochemical techniques including FTIR, UV-Vis, NMR, mass spectrometry, conductivity measurements, and thermal analysis are employed to identify the products. The produced materials are examined for their dyeing properties and biological activities. This is followed by calculations based on density functional theory (DFT) and time-dependent DFT (TD-DFT) to validate their proposed structures and properties. Additionally, molecular docking is conducted on the products exhibiting high efficiency as bioactive agents against *S. aureus* and *E. coli*.

## Methodology

### Materials and measurements

All used materials and physicochemical measurements are described in detail in the supplementary file (S1 materials and measurements).

### Dyeing properties study

#### Dyeing process

While preparing the dye-bath, 2.0 g of fabric samples were added to a flask along with a 2% (o.w.f) dye shade and dispersing agent. The flask was then heated to 130 °C for one hour with a 1:50 liquor ratio. Acetic acid was used to bring the pH down to 4.5 [43]. Once the dyeing procedure was complete, the colored samples were removed and exposed to a temperature of 60 °C for 10 min to relieve in reduction cleaning. After that, they were rinsed with tap water and allowed to dry naturally.

#### Color fastness to rubbing

The ISO 105-X12:1987 test method measured color fastness against rubbing [44]. To determine how much color from colorful textiles can transfer to another surface through rubbing. Dry and wet fabrics can be used for this study. A dry crocking test was done by placing the fabric flat on the crock meter base. A white test cloth was affixed. The test specimen glided 20 times on the covered finger. The white test sample was then removed and stained with grey scale for examination. After extraction, the white test sample was colored grey. Wet crocking was performed on 65% saturated white test samples. As before, the technique was used. Air-dried test samples preceded analysis.

#### Color fastness to perspiration

Following the guidelines provided by ISO 105-E04:1989, two synthetic perspiration solutions; one acidic and one alkaline were made [45]. Mixing 0.5 g of L-histidine monohydrochloride monohydrate, 5.0 g of NaCl, and 2.2 g of NaH_2_PO_4_.2H_2_O in 1 L of distilled water produced the acidic solution. The pH was then brought down to 5.5 using 0.1 N NaOH. Similarly, the alkaline solution was prepared by dissolving 0.5 g of L-histidine monohydrochloride monohydrate, 5.0 g NaCl, and 2.5 g of Na_2_HPO_4_.2H_2_O into 1 litter distilled water, to form the alkaline solution. Using 0.1 N NaOH, the pH was brought down to 8. What followed was the procedure for the fastness test. A 5 cm×4 cm colored sample was stitched between two 4 cm × uncolored samples to create a composite specimen. To ensure complete saturation, the composite samples were submerged in both solutions for 15–30 min, agitated vigorously, then compressed. The specimens under examination were forcefully clamped between two plastic or glass plates using a force of about 4 to 5 kg. The composite samples were kept in an oven at a temperature of 37 ± 2 °C for four hours after being arranged vertically on plates. The specimens that were analyzed had their color modification assessed and reported using a grey scale.

### Biological activity

The In vitro antimicrobial activities, cytotoxicity screening and antioxidant properties of the studied compounds were recorded, and detailed procedures are explained in the supplementary file; Methods S1, S2, and S3. The in vitro antimicrobial activities were screened against pathogenic Gram-positive bacterial strains; *Staphylococcus aureus*(RCMB 010010) and *Bacillus subtilis*(RCMB 015 (1) NRRL B-543) and, Gram-negative bacterial strains; *Escherichia coli*(RCMB (010052) ATCC 25955), *proteus vulgaris* (RCMB 004 (1) ATCC 13315) in addition to pathogenic fungi *Aspergillus flavus* (RCMB 002008) and *Candida albicans*(RCMB 005003 (1) ATCC 10231). It worth to mention that all microbial strains were provided from culture collection of the Regional Center for Mycology and Biotechnology (RCMB), Al-Azhar University, Cairo, Egypt. Antimicrobial activities of the tested samples were determined using a modified Kirby-Bauer disc diffusion method [46, 47]. The in vitro cytotoxicity screening was applied to the mammalian cell lines: A-549 cells (human Lung cancer cell line). The cells were obtained from VACSERA Tissue Culture Unit [48, 49]. The antioxidant activities were determined *by* the DPPH free radical scavenging assay in triplicate and average values were considered [50].

### Synthesis of 4-((3-formyl-4-hydroxyphenyl)diazenyl)benzenesulfonamide, (HL)

The synthesis procedure started with dissolving sulfanilamide (1.722 g, 10 mmol) in 20 mL, 34% hydrochloric acid and 20 mL distilled water, cooling in ice bath with constant stirring, then dropwise addition of a cold solution of sodium nitrite (0.69 g, 10 mmol), and temperature was adjusted in the range (0–5)^o^C. The diazonium salt was added dropwise to a solution of salicylaldehyde (1.05mL, 10 mmol) in 1.2 M NaOH (50 mL), while adjusting the pH of solution at 5.0-5.5 by addition solid NaHCO_3_ with constant stirring [51, 52]. The yellow solid precipitated was filtered, washed several times with distilled water and ethanol, then dried under vacuum at 60 °C for 24 h.

HL; C_13_H_11_N_3_O_4_S; yellow solid, %yield: (48%), m.p:139^o^C, m/z; 305.05, elemental analysis (calc./found.): C; 10.50(10.39), H; 3.63(3.77), N; 13.76(13.92), S; 51.14(51.04).

FTIR spectra (KBr, cm^− 1^); ʋ(C = O) 1666 (vs.), ʋ(C-O) phenolic 1280, ʋ(N = N)azo 1481, ʋ_asym_(S = O) 1334, ʋ_sym_(S = O) 1157.

UV–vis. (DMSO, λ (nm), ε (L mol^− 1^ cm^− 1^)); (n- π*) 355 (11168) and 480(1879).

^1^H-NMR δ (ppm): 11.76 (1H, OH), 10.37 (1H, CHO), 8.21–7.25 (7 H, aromatic protons), 7.37 (1H, NH_2_). ^13^C NMR (126 MHz,) 190.33 (C = O), (164.04, 153.39, 131.83, 129.96, 128.97, 127.09, 125.60, 124.20, 122.79, 118.61 aromatic carbons).

### Synthesis of Schiff bases; 4-((4-hydroxy-3(((2-hydroxyphenyl)imino)methyl) phenyl)diazenyl)benzenesulfonamide)], (HL^1^) and 4-((4-hydroxy-3-(((2-mercaptophenyl) imino)methyl)phenyl)diazenyl)benzenesulfonamide), (HL^2^)

Schiff bases HL^1^ and HL^2^ were synthesized by mixing a hot ethanolic solutions of HL (1.5 g, 5mmole/30mL) and 2-aminophenol (0.6 g, 5mmol/20mL) or 2-aminothiophenol (0.55mL, 5mmole/20mL). Reaction mixtures were subjected to reflux at 80 ^o^C for 12 h. The formed solids were filtered off, washed with distilled water and ethanol, followed by drying in vacuum at 60^o^C for 24 h.

HL^1^; C_19_H_16_N_4_O_4_S, orange solid, %yield; 43.43%, m.p. 253 ^o^C. m/z; 395.85. elemental analysis (calc./found.): C; 57.57(57.61), H; 4.07(3.96), N; 14.13(14.01), S; 8.09(8.24).

FTIR spectra (KBr, cm^− 1^); ʋ(OH) 3425, ʋ(C = N)_azomethine_ 1612 (vs.), ʋ(C-O) 1303, 1249, ʋ(N = N)azo 1465, ʋ_asym_(S = O) 1334, ʋ_sym_(S = O)1149.

UV–vis. (DMSO, λ (nm), ε (L mol^− 1^ cm^− 1^)); (n- π*) 355 (1471) and 480(4438).

^1^H-NMR δ (ppm): 10.17 (1H, OH), 9.24 (1H, CH = N), 8.28–6.94 (11 H, aromatic protons).

^13^C NMR (126 MHz,) 168.77 (CH = N), (168.77, 159.56, 153.31, 150.38, 144.72, 143.17, 131.39, 130.52, 128.42, 126.74, 126.67, 122.17, 119.74, 119.44, 118.64, 117.84, 116.24 aromatic carbons).

HL^2^, C_19_H_16_N_4_O_3_S_2_; brown solid, %yield 41.46%, m.p. 200 ^o^C. m/z; 412.62. elemental analysis (calc./found.): C; 55.32(55.93), H; 3.91(3.79), N; 13.58(13.87), S; 15.55(15.41).

FTIR spectra (KBr, cm^− 1^); ʋ(OH) 3325, ʋ(C = N)_azomethine_ 1612 (vs.), ʋ(C-O) 1273, ʋ(S-H) 1921, ʋ(C-S) 694, ʋ(N = N)azo 1489, ʋ_asym_(S = O) 1327, ʋ_sym_(S = O)1149.

UV–vis. (DMSO, λ (nm), ε (L mol^− 1^ cm^− 1^)); (n- π*) 325 (5692), 345 (4967), 390 (4949) and 515 (5213).

^1^H-NMR δ (ppm): 12.39 (1H, SH), 11.61 (1H, OH), 8.88 (1H, CH = N), 8.16–7.32 (11 H, aromatic protons). ^13^C NMR (126 MHz,) 162.63 (CH = N), (159.28, 153.20, 151.08, 145.12, 144.70, 134.75, 126.75, 126.52, 126.20, 124.88, 123.55, 122.46, 122.12, 121.74, 119.38, 117.59 aromatic carbons).

### Synthesis of the Cu(II) and Zn(II) complexes of HL^1^ and HL^2^ (general method)

Metal complexes of HL^1^ and HL^2^ ligands were synthesized by the addition of a warm ethanolic or methanolic solution (20mL) CuCl₂.2H₂O (0.34 g, 2mmol) or (0.17 g, 1mmol) and Zn(CH₃COO)₂.2H₂O (0.44 g, 2mmol) or (0.22 g, 1mmol), was added to the appropriate organic ligands HL^1^ (0.79 g, 2mmol) or HL^2^ (0.41 g, 1mmol) dissolved in the same solvent (30mL). The mixture was left under reflux with continuous stirring for 12 h at 70–80 °C, and the precipitates were collected by filtration, washed by cold ethanol, and dried at room temperature.

Bis-2-((2-hydroxy-4-((4-sulfamoylphenyl)diazenyl)benzylidene)amino)phenolato-copper(II)pentahydrate, [Cu(L^1^)₂]0.5 H₂O, C_38_H_40_CuN_8_O_13_S_2_: Brown Solid, m.p. >300 ^o^C, m/z; 943.15, molar conductivity 32.5 × 10^− 5^ Ω^−1^cm^2^mol^−1^, elemental analysis (calc./found.): C; 48.33 (48.57), H; 4.27 (4.31), N; 11,86 (11.94), S; 6.79 (6.58), Cu; 6.73 (6.69).

FTIR spectra (KBr, cm^− 1^): ʋ(C = N) 1604, ʋ(N = N) 1473, ʋ(C-O) 1303, 1275, ʋ_asym_(S = O) 1334, ʋ_sym_(S = O) 1157.

UV–vis. (DMSO, λ (nm), ε (L mol^− 1^ cm^− 1^)); (n- π*) 440 (10296), 475 (13749) and 505 (10296).

Bis-2-((2-hydroxy-4-((4-sulfamoylphenyl)diazenyl)benzylidene)amino)benzene-thiolatocopper(II)trihydrate, [Cu(L^2^)₂]0.3 H₂O, C_38_H_36_CuN_8_O_9_S_4_: Olive green solid, m.p. >300 ^o^C, m/z; 939.08, molar conductivity 19.9 × 10^− 5^ Ω^−1^cm^2^mol^−1^, elemental analysis (calc./found.): C; 48.53 (48.73), H; 3.86 (3.65), N; 11.91 (12.20), S; 13.64 (13.51), Cu; 6.76 (6.81).

FTIR spectra (KBr, cm^− 1^): ʋ(C = N) 1604, ʋ(N = N) 1489, ʋ(C-O) 1273, ʋ(C-S) 678, ʋ_asym_(S = O) 1327, ʋ_sym_(S = O) 1157.

UV–vis. (DMSO, λ (nm), ε (L mol^− 1^ cm^− 1^)); (n- π*) 325 (18825), 350(16700), 385(15145) and 505 (12335).

Bis-2-((2-hydroxy-4-((4-sulfamoylphenyl)diazenyl)benzylidene)amino)phenolato-zinc(II)dihydrate, [Zn(L^1^)₂].2H₂O, C_38_H_34_N_8_O_10_S_2_Zn: Dark orange solid, m.p. >300 ^o^C, m/z; 890.11, molar conductivity 5.2 × 10^− 5^ Ω^−1^cm^2^mol^−1^, elemental analysis (calc./found.): C; 51.15 (51.46), H; 3.84 (3.72), N; 12.56 (12.24), S; 7.19 (7.36), Zn; 7.33 (7.0).

FTIR spectra (KBr, cm^− 1^): ʋ(C = N) 1604, ʋ(N = N) 1473, ʋ(C-O) 1296, 1257, ʋ_asym_(S = O) 1334, ʋ_sym_(S = O) 1149.

UV–vis. (DMSO, λ (nm), ε (L mol^− 1^ cm^− 1^)); (n- π*) 380 (27810) and 460 (49520).

^1^H-NMR δ (ppm): 9.67 (2(1H, CH = N)), 8.82–7.14 (2(11 H, aromatic protons)).

Bis-2-((2-hydroxy-4-((4-sulfamoylphenyl)diazenyl)benzylidene)amino)benzene-thiolatozinc(II)dihydrate, [Zn(L^2^)₂].2H₂O, C_38_H_34_N_8_O_8_S_4_Zn: Dark brown solid m.p. >300, m/z; 922.07, molar conductivity 13.6 × 10^− 5^ Ω^−1^cm^2^mol^−1^, elemental analysis (calc./found.): C; 49.38 (49.16), H; 3.71 (3.92), N; 12.12 (12.37), S; 13.88 (13.69), Zn; 7.07 (6.90).

FTIR spectra (KBr, cm^− 1^): ʋ(C = N) 1604, ʋ(N = N) 1496, ʋ(C-O) 1273, ʋ(C-S) 678, ʋ_asym_(S = O) 1334, ʋ_sym_(S = O) 1157.

UV–vis. (DMSO, λ (nm), ε (L mol^− 1^ cm^− 1^)); (n- π*) 405 (18930) and 505 (27230).

^1^H-NMR δ (ppm): 8.76 (2(1H, CH = N)), 7.96–6.47 (2(11 H, aromatic protons)).

### Computational methodology

A large range of synthesized transition metal complexes structures were interpreted with density functional theory (DFT) leading to investigation of the geometrical and electronic structures of complicated molecules [53, 54]. In order to conduct theoretical calculations, the Gaussian 09 software [55] was utilized, which included the application of Becke’s three parameter exchange Lee-Yang-Parr correlation functional (B3LYP) with the basis set LANL2DZ [56, 57]. Firstly, full optimization was performed on Cu(II) complexes 1 and 2, Zn(II) complexes 1 and 2, then analysis of vibrational frequencies to indicate that the optimized structures at stationary points corresponding to minima without imaginary frequencies. While in the gaseous state, geometrical data including bond lengths and bond angles were computed. Some important quantum and reactivity parameters such energies of highest molecular orbital and lowest unoccupied molecular orbital, ionization potential, electron affinity, electronegativity and dipole moment were calculated such as optimization energy, reactivity parameters and HOMO, LUMO energies. Gauss view [58] and Chem craft programs [59] helped in visualize the optimized structures and in extraction of some calculation results the frontier molecular orbitals (FMOs). Infrared absorption and electronic absorption spectra of the optimized structure were calculated in gas phase and in ethanol. Electronic spectra were calculated applying (TD-DFT)/B3LYP method with LANL2DZ basis set in gas phase [60, 61].

### Molecular docking study

Selecting an antimicrobial target protein by evaluating the potential of ligand affinity against competing docked positions and applying appropriate docking parameters [62, 63]. The 6QF6-, and 5ND9- macromolecular crystal structures of negative bacteria (*Escherichia coli*) and positive bacteria (*Staphylococcus aureus*) were investigated by Luptak, J [64]. and Khusainov, I. et al. [65], respectively. These structures were obtained from the Protein Data Bank website (https://www.rcsb.org/) in order to examine a range of protein complexes and their activity. The optimized complexes have been docked within the active region of the bacterial protein as a receptor to examine the intensity of the contact taking place. iGemdock 2.1 program was used to study the molecular docking behavior [66]. Prior to the investigation, the selected target protein was prepared by removing any excess ions, ligands, and water molecules that were not relevant to the study protocol. Polar hydrogens were added to the proteins and Gasteir charges were assigned to them. In docking accuracy settings, genetic algorithms (GA parameters) are employed with a population size of 200 and a selection of 70 generations, each with multiple solutions equal to two. The Chimera 1.13.1 [67] software is used to visualize and define the types of nonbonding interactions that arise from the docking tool.

## Results and discussions

**Scheme 1:**
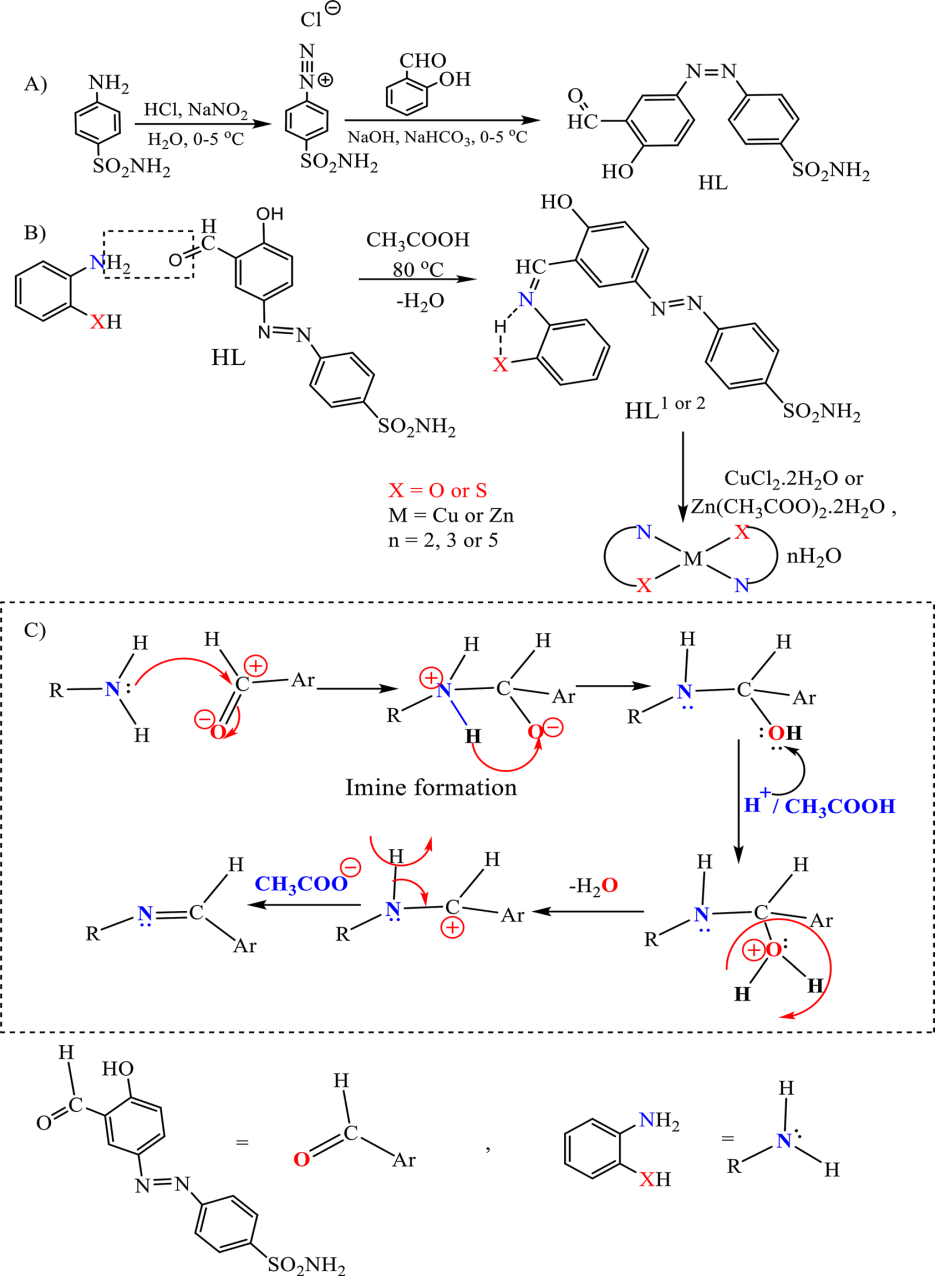
Synthesis of free ligands and their complexes

**Scheme 2:**
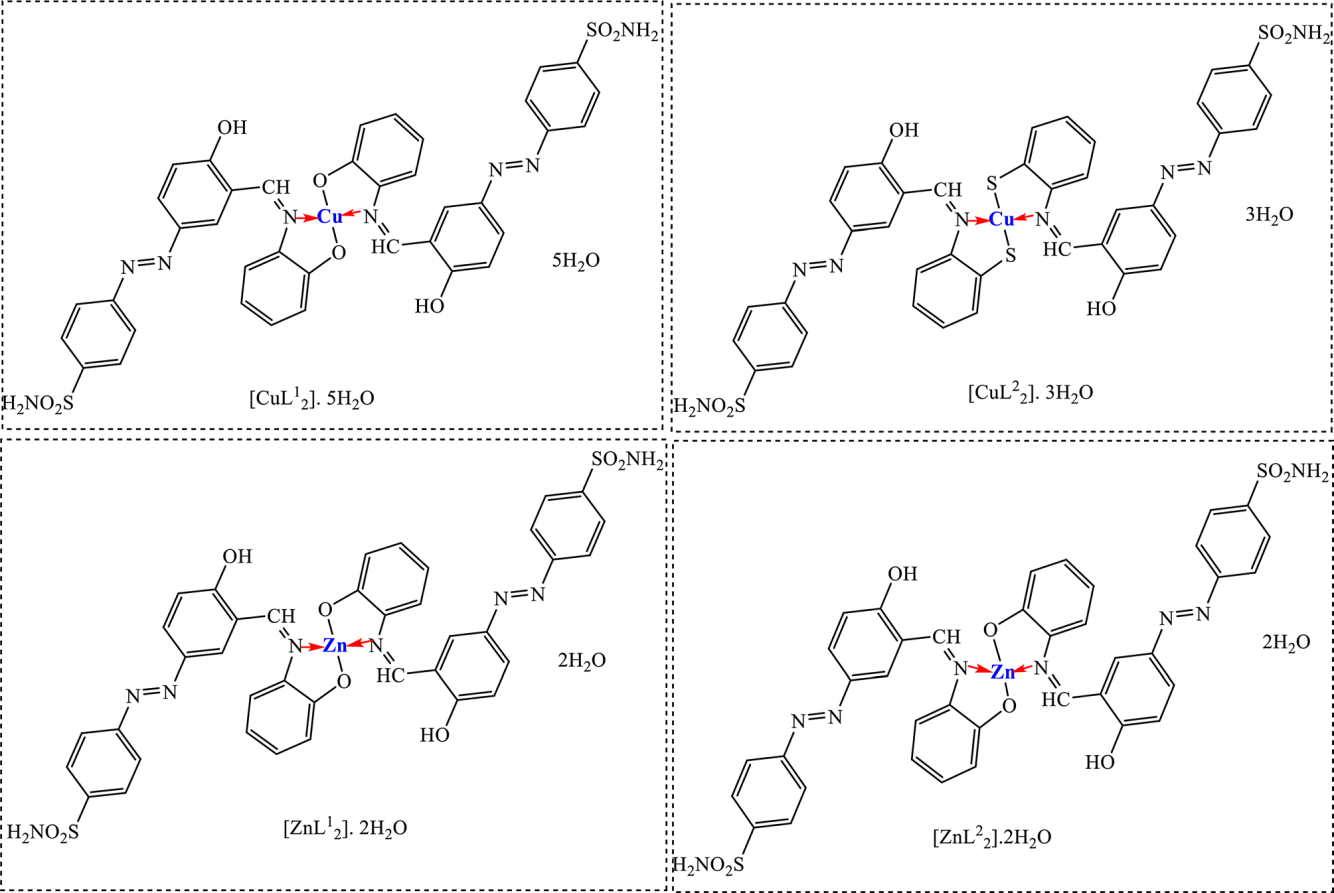
Proposed structures of Cu(II) and Zn(II) complexes

The condensation of the HL with *o*-aminophenol and *o*-amino-thiophenol gives the corresponding azo-azine compounds abbreviated as HL^1^ and HL^2^, respectively. Scheme (1) presents: (A) the formation of azo derivatives, (B) the azo Schiff bases, which are formed in presence of acetic acid assisting the formation of imine group C = N, and their interaction with Cu(II) and Zn(II) to form metal complexes, (C) the mechanism of imine group formation. Scheme (2) represents the proposed structures of the synthesized metal complexes.

The compounds exhibit long-term air stability at ambient temperature. The azo compound (HL) is fully soluble in MeOH, EtOH, DMF, and DMSO, sparingly soluble in acetonitrile but insoluble in H_2_O, while the azo Schiff bases (HL^1^ and HL^2^) are soluble in DMF and DMSO, sparingly soluble in MeOH, EtOH but insoluble in H_2_O. The molar conductivity values of 1.0 × 10^− 3^ M- DMF solutions, at 25 °C ± 1 of Cu(II) and Zn(II)metal complexes were found to be very low indicating their non-electrolytic nature. The yellow color of the HL is changed into orange and brown colors upon Schiff base formation with *o*-aminophenol and *o*-aminothiophenol, respectively. This might be attributable to the greater conjugation following Schiff base formation and the softer nature of the sulfur atom compared to the oxygen atom.

### Characterization of free ligands and their metal complexes

The ligands (HL, HL^1^, and HL^2^) and their metal (Cu(II) and Zn(II)) complexes, were isolated as powder solids, Schemes (1 and 2). The isolation of individual crystals was unsuccessful, regrettably. Thus, a number of physicochemical techniques were employed, including infrared (IR), nuclear magnetic resonance (NMR), ultraviolet (UV) spectroscopy, electrospray spectroscopy (ESR), mass spectroscopy, elemental analysis, and molar conductivity, to elucidate the structures of products. The structures of the products were also supported by computational investigations.

#### FT-IR spectral studies

**Fig. 1:**
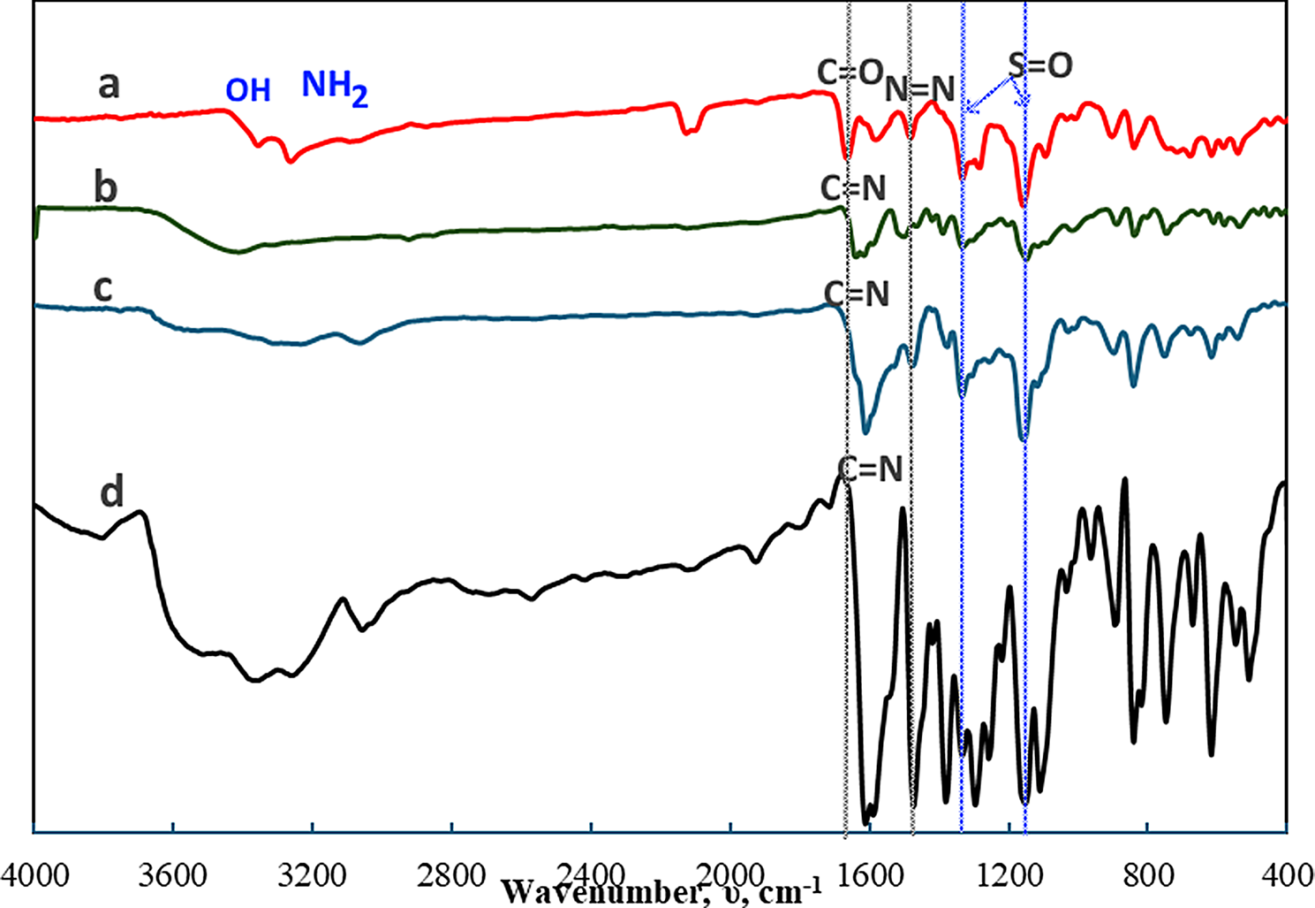
FT-IR spectra of a) HL, b)HL^1^, c) [CuL^1^_2_].3H_2_O, and d)[ZnL^1^₂]0.2 H₂O

**Fig. 2:**
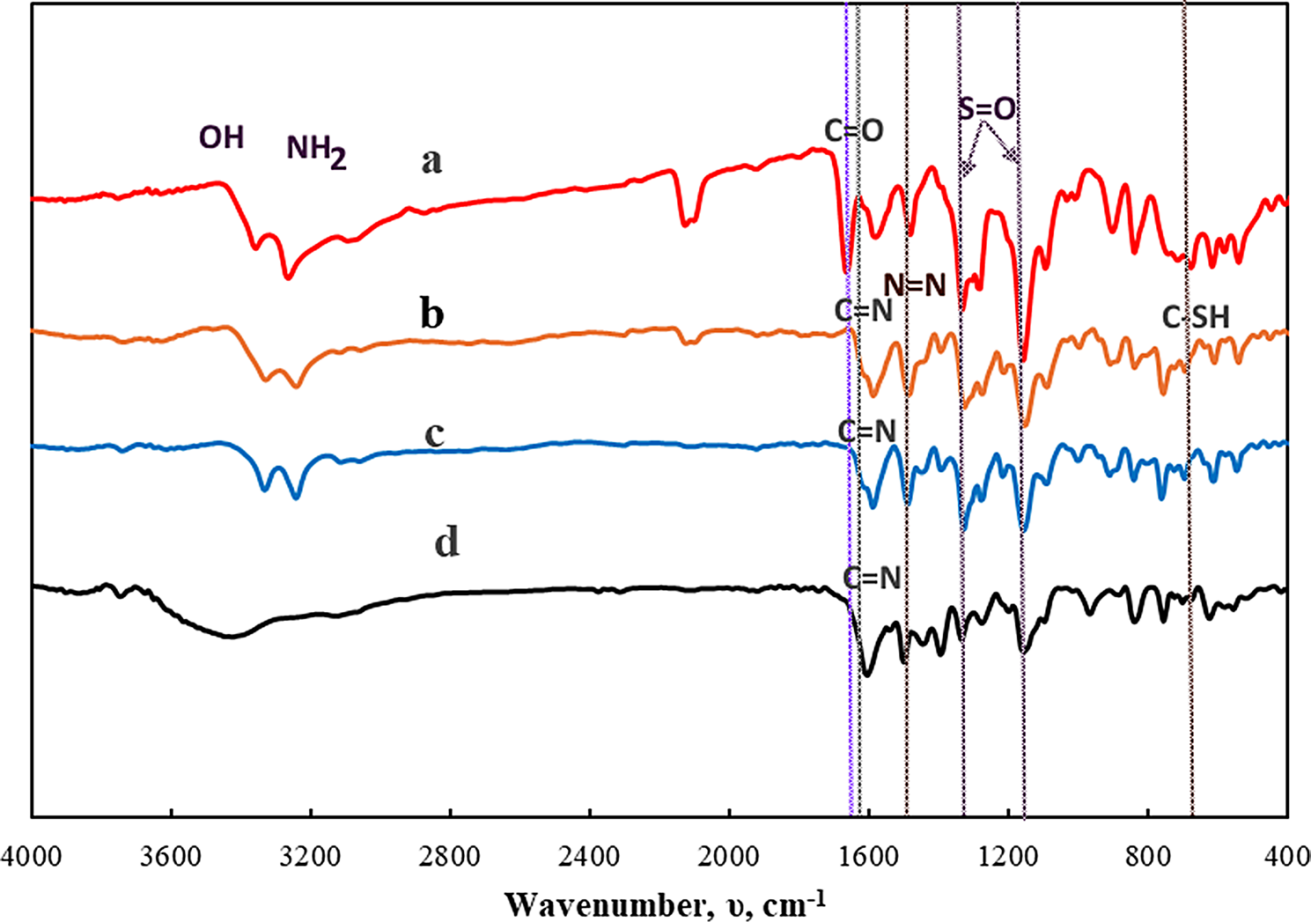
FT-IR spectra of a) HL, b) HL^2^, c) [CuL^2^_2_].3H_2_O, and d)[ZnL^2^₂].2H₂O

The FT-IR spectra of synthesized organic compounds; HL, HL^1^ and HL^2^ and their metal complexes are recorded as KBr discs in range of 400–4000 cm⁻¹, Figs. 1 and 2. By comparing the spectra of the three designated organic compounds, it is possible to discern the absence of the aldehydic group (C=O), which was observed in the HL as a distinct, intense band at 1666 cm^− 1^ [68], and the appearance of a new band at 1612 cm^− 1^ in case of HL^1^ and HL^2^ which is ascribed to the azomethine group band (C=N) [69]. In addition, ʋ(C-O) of the phenolic OH appeared as one medium sharp band at 1280 and 1273 cm^− 1^, in HL and HL^2^, respectively [70], whereas in case of HL^1^, the ʋ(C-O) of the salicylaldehyde and aminophenol moieties showed two bands at 1303 and 1249 cm^− 1^, respectively. The appearance of a medium sharp band at 1481,1465 and 1489 cm^− 1^ is assigned to ʋ(N=N) of the azo group for HL, HL^1^ and HL^2^, respectively. The bands at 1334 and 1157 cm^− 1^ are attributed to asymmetric and symmetric ʋ(S=O), respectively [71]. The spectra also show bands at 3267–3368 cm^− 1^ corresponding to ʋ(NH_2_) of sulfanilamide and phenolic ʋ(OH) [72, 73]. However, the spectrum of HL^2^ displayed a weak band at 1921 cm^− 1^ due to ʋ(S-H) which is confirmed by the presence of a weak band at 694 cm^− 1^ attributed to ʋ(C-S) of thiophenol group [74]. It worth to mention that the spectra of HL^1^ and HL^2^ exhibits red shifts for the band characteristic of ʋ_sym_(SO_2_) while the band due to ʋ_asym_(SO_2_) remained unchanged in case of HL^1^ only.

The FT-IR spectra of Cu(II) and Zn(II) complexes play a role in understanding HL^1^ and HL^2^ ligand bonding. In case of HL^1^ complex formation, the participations of azomethine and phenolic oxygen moieties in coordination to metal centers are indicated from the red shifting of the corresponding bands at 1612 cm^− 1^ and 1249 cm^− 1^ by 8 cm^− 1^. Furthermore, the characteristic band of ʋ(C-O) phenolic of the salicylaldehyde at 1303 cm^− 1^ in the free HL^1^ appeared either at the same position in Cu(II) complexes or red shifted to 1296 cm^− 1^ in Zn(II) complexes. This extent of shift could be taken as evidence for the involvement of the OH of the salicylaldehyde in an intra-or inter-molecular hydrogen bonding. However, there is a small shift the band of ʋ(N=N) at 1465 cm^− 1^ in the free HL^1^ was red shifted to 1470 cm^− 1^ in the complexes without changing its shape suggesting its non-bonding nature to metal ion and could be referred to the electronic effects resulted from the complexation through the phenolic OH and azomethine-N. The spectra of the complexes of HL^2^ revealed a disappearance of the band characteristic to ʋ(S-H) at 1921 cm^− 1^, indicating its deprotonation upon complex formation. The participation of the sulfur atom in bonding to the metal ion maybe recognized from the red shift of ʋ(C-S) from 694 cm^− 1^ in the free HL^2^ to 671–678 cm^− 1^ in complexes. The spectra of [L^2^_2_M], M = Cu(II) or Zn(II), exhibit a red shift for the ʋ(C = N) band from 1612 cm^− 1^ in the free HL^2^ to 1604 cm^− 1^. This shift could be taken as evidence for the bonding nature of the azomethine-N to the metal ion. The non-bonding nature of the -N = N- in the complexes is confirmed by its appearance in the same position (1489 cm^− 1^) in case of Cu(II) complex, while a small shifted to 1496 cm^− 1^ in case of Zn(II) complex, that may result from the different electronic and conformation effects. The spectra of the studied metal complexes display a small blue shift or no shift for the ʋ_asym_(SO_2_) and ʋ_sym_(SO_2_). This observation suggests the non-bonding nature of this group to the metal ion and the formulation of the ligand.

Accordingly, the CHN and FT-IR data confirmed the proposed stoichiometry and structure of the prepared compounds, Scheme (1).

#### Electronic spectra

**Fig. 3:**
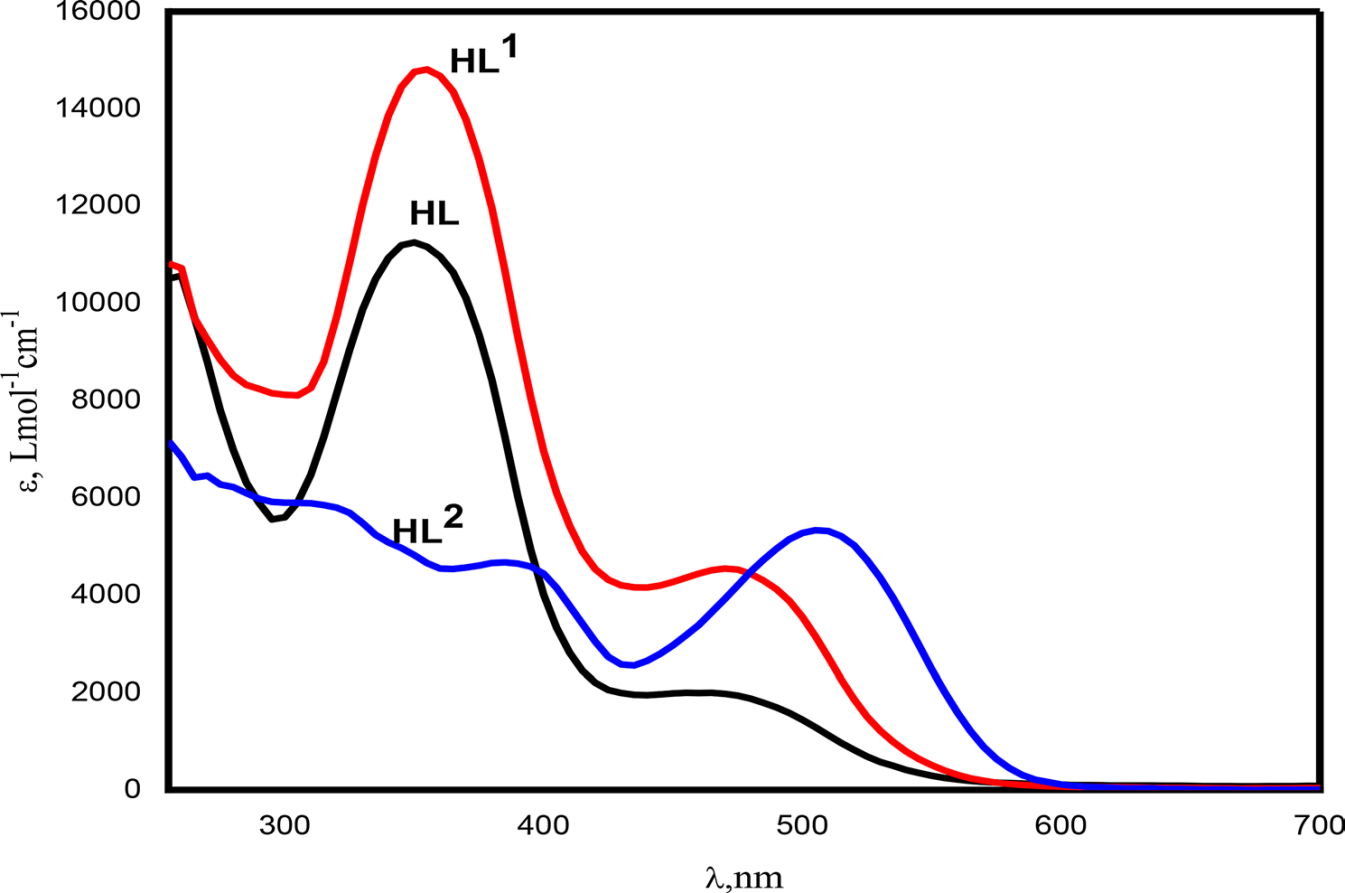
Room temperature electronic spectra of HL, HL^1^ and HL^2^ in DMF as solvent

**Fig. 4:**
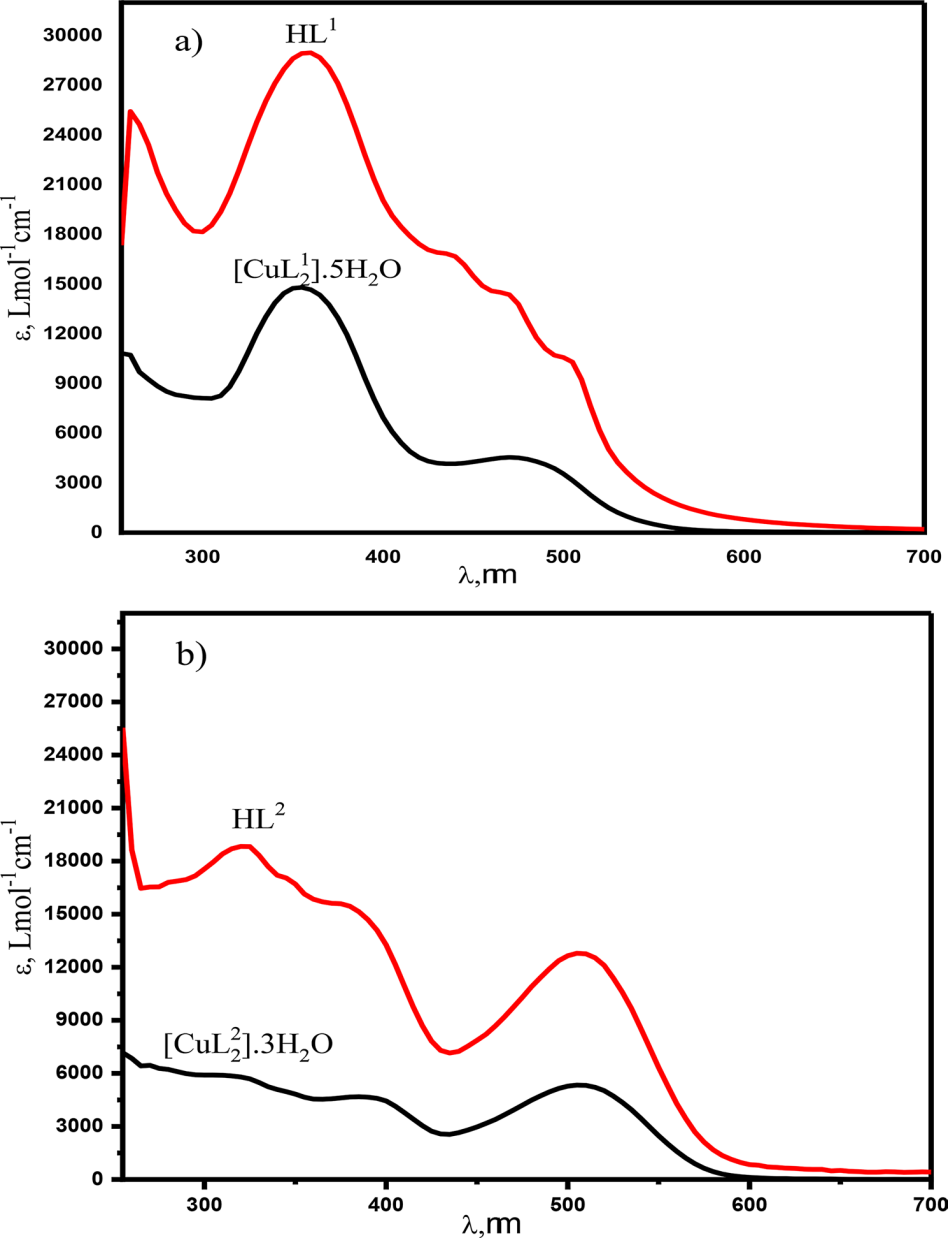
Room temperature electronic spectra of **a**) HL^1^ and its copper(II) complex and **b**) HL^2^ and its copper(II) complex in DMF solvent

**Fig. 5:**
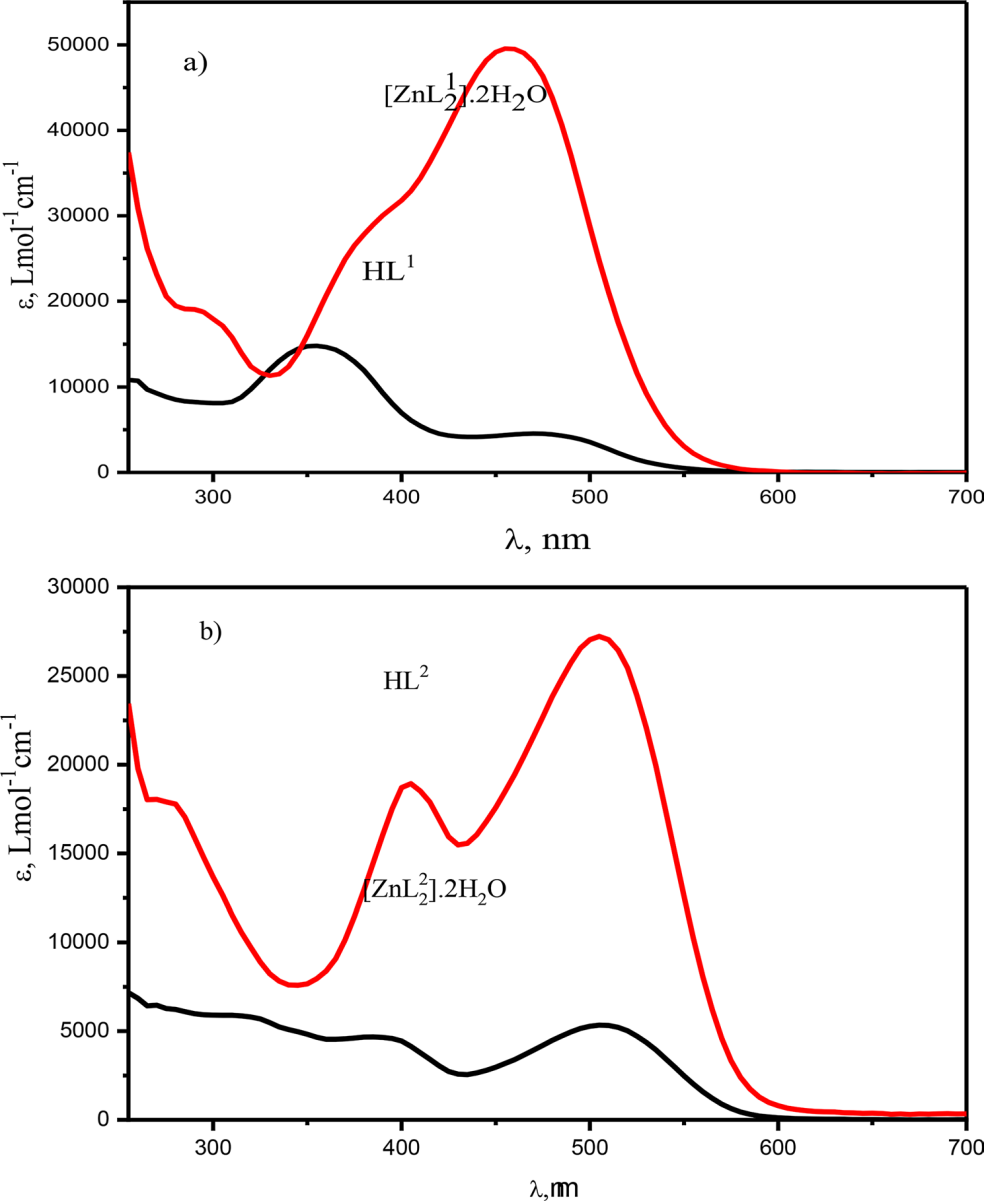
Electron spectra of **a**) HL^1^ and its Zn(II) complex and **b**) HL^2^and its Zn(II) complex in DMF solvent

The electronic absorption spectra of the synthesized compounds were recorded as 1.00 × 10^− 3^ − 1.00 × 10^− 5^ M DMF solutions in the range of 250–1000 nm, Figs. 3 and 4. The azo compound HL and its azo-Schiff bases HL^1^ and HL^2^ exhibit strong bands (250–400 nm) due to intermediate energy π- π* transitions of aromatic rings, azo, and azomethine groups [70]. The absorption band between 400 and 500 nm is caused by n-π* transitions in azo and azomethine chromophores. These transitions coincide with charge transfer transitions occurring in the azo-Schiff complexes. The intramolecular charge transfer band may be attributed to the potential tautomeric equilibrium arising from the hydroxyl group in the o-position of the aromatic ring. The spectra of HL and HL^1^ had two n- π* transition bands at 355 and 480 nm, with distinct molar absorptivity, the higher energy band has ɛ values of 11,168 and 14,671 Lmol^− 1^cm^− 1^, while the lower energy one has ɛ values of 1879 and 4438 Lmol^− 1^cm^− 1^ for HL and HL^1^, respectively. On the other hand, the spectrum of HL^2^ exhibits four bands at 325 nm (ɛ = 5692 Lmol^− 1^cm^− 1^), 345 nm (ɛ = 4967 Lmol^− 1^cm^− 1^), assigned to n- π * and 390 nm (ɛ = 4949 Lmol^− 1^cm^− 1^) and 515 nm (ɛ = 5213 Lmol^− 1^cm^− 1^) that may attributed to the charge transfer transitions. The azo-Schiff bases are rich as ligating agents toward metal ions since they contain π-acceptor sulfur atoms, π-donor oxygen atoms, in addition to azomethine nitrogen. In case of [CuL^1^_2_(H_2_O)_2_]. 3H_2_O, Fig. 4a, the broad band centered at 480 nm(ɛ = 4438 Lmol^− 1^cm^− 1^) of the ligand HL^1^ has split into three bands at 440 nm (ɛ= 10296 Lmol^− 1^cm^− 1^), 475 nm (ɛ= 13794 Lmol^− 1^cm^− 1^) and 505 nm (ɛ= 10296 Lmol^− 1^cm^− 1^) showing higher molar absorptivity, whereas [CuL^2^_2_(H_2_O)_2_]. H_2_O showed blue shifted bands from 390 to 515 nm in the free ligands to 385 and 505 nm, respectively, Fig. 4b. The band at 345 nm in the free ligand is red shifted to 350 nm, while the band at 325 nm in the free ligand remained unchanged. The ɛ values for all showed increments. However, these spectral features and changes can be taken as evidence for the complex formation and contribution of the intra ligand charge transfer and LMCT transitions. On the other hand, the electronic absorption spectra of the products resulting from the interaction of Zn(II) with HL^1^ or HL^2^ showed red shift of azomethine n-π* transition band at 355 and 345 nm of the free ligand to 380 and 405 nm with increased intensity upon complexation, respectively, whereas the bands at 480 and 515 nm, assigned to the intra-ligand (CT) and the azo n-π* transitions, are blue shifted to 460 and 505 nm with increasing intensity for the Zn(II) complexes of HL^1^ and HL^2^, respectively, Fig. 5.

#### NMR spectra

The ^1^H-NMR and ^13^C-NMR spectra of HL^1^, HL^2^ and their Zn(II) complexes as d^6^-DMSO solutions, displayed signals that were consistent with the proposed structures, Figures S1 & S2. The low field positions of most signals in the^1^H-NMR spectra of free ligands and their Zn(II) complexes may referred to the formation of intramolecular (OH…O = CH) and/or (OH…N=CH), intermolecular (OH…DMSO), (DMSO…H_2_NSO_2_) as well as intermolecular hydrogen bonding between the ligand molecules. The ^1^H-NMR spectra of HL^1^ display signals at δ 10.24 (2H), 9.25 (1H) and 8.28–6.91 (13H) ppm due to (OH, 1 H) proton, which does not completely exchange when D_2_O is added to d_6_-DMSO solution, azomethine (CH=N) proton and amino (NH_2_) proton which overlapped with aromatic protons, respectively [75, 76]. However, the ^13^C NMR spectrum of the ligand exhibits signals attributed to aromatic carbons (δ 159.81–116.60 ppm) and azomethine carbon (δ 169.25 ppm). In the case of Zn(II) complexes, the signal at δ 10.24 ppm vanished entirely, while the signals of aromatic and -NH_2_ protons (8.28–6.91 ppm) (26 H), as well as azomethine protons (δ 9.25 ppm) (2H), were down field-shifted and emerged at δ 9.67 and 0.885-7.20 ppm, respectively. These findings point to the deprotonation of one phenolic OH during complex formation, with the other potentially being transferred to DMSO, which would explain why it appeared outside of the experimental range. Thus, the data support the coordination of HL^1^ to Zn(II) through azomethine-N and phenolate-O atoms.

The ^1^H-NMR spectra of HL^2^ in d_6_-DMSO showed a broad signal at δ12.48 ppm (2H) corresponds to the SH and OH protons, which disappeared upon adding D_2_O to the d_6_-DMSO solution. The downfield shift of signals may be attributed to its participation in specific types of hydrogen bonding. The signal at δ8.90 ppm corresponds to the azomethine proton (CH=N). The NH_2_ and aromatic protons signals showed up at δ8.17–7.43 (13H) ppm [77]. The ^13^CNMR of HL^2^ showed an azomethine carbon signal at δ 162.82 ppm and aromatic carbon atoms signals ranging from 159.77 to 117.94 ppm. The ^1^H-NMR spectra of [Zn(L^2^)_2_].2H_2_O showed the aromatic and NH_2_ protons with chemical shifts of δ 7.97–6.46 ppm (26 H). The increase in the field shift from the free HL^2^ can be attributed to the shielding effect during complexation. The CH=N proton signal (2H) is moved up field to δ8.76 ppm, indicating its shielding following attaching to Zn(II). The absence of SH and OH proton signals may be attributed to their deprotonation when attaching to Zn(II) and interacting with DMSO, respectively.

#### Electron paramagnetic resonance spectra (EPR) of Cu(II) complexes

**Fig. 6:**
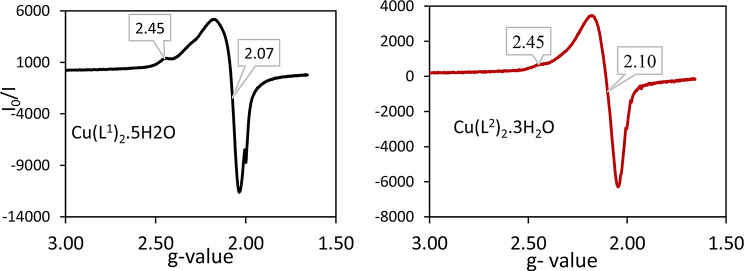
X-band EPR spectrum of the polycrystalline of Cu(L^1^)_2_.5H_2_O and Cu(L^2^)_2_.3H_2_O, at room temperature

The room temperature X-band EPR spectra of the polycrystalline [Cu(L^x^)_2_(H_2_O)_2_].nH_2_O, where L^x^= L^1^ or L^2^ are shown in Fig. 6. The spectra were found to be of axial shape with g_||_ = 2.45 > g_┴_= 2.07 and 2.10 for L^1^ or L^2^, respectively. The values of g_av_ are characteristics of species with more populated in the ground state [78]. The high values of g_||_ in both complexes (2.45) indicate the ionic character of metal-ligand bond. The calculated G- values are found to be 6.61 and 4.58 for L^1^ and L^2^ complexes, respectively, in agreement with the absence of a magnetic exchange interaction between the copper centers in the solid state [79, 80].

#### Mass spectra of free ligands and their metal complexes

For the sake of affirmation of the proposed formulae and molecular weights of free ligands and their metal complexes, the mass spectra of synthesized compounds are recorded utilizing electron impact mass spectrometry (EI), shown in Figures (S3 and S4), and assumed fragmentations are illustrated in Schemes (S1-S7). However, the molecular ion peaks *m*/*z*, showed high agreements with proposed molecular formulae, the observed values for HL, HL^1^ and HL^2^ are 305.20, 395.85 and 412.62, corresponding to (C_13_H_11_N_3_O_4_S, F.W. 305.05), (C_19_H_16_N_4_O_4_S, F.W. 396.09) and (C_19_H_16_N_4_O_3_S_2_, F.W. 412.07), respectively. The spectra of copper(II) complexes confirmed the proposed formulae and agreed with the calculated molecular weights as [Cu(L^1^)₂]0.5 H₂O, exhibited a molecular ion peak at m/z 943.12 (calculated as 943.15) and [Cu(L^2^)₂]0.3 H₂O was found as 939.33, calculated as 939.08. The mass spectra of [Zn(L^1^)₂].2H₂O and [Zn(L^2^)₂].2H₂O, also showed parent peaks at m/z 890.60 and 922.66 corresponding to (C_38_H_34_N_8_O_10_S_2_Zn, 890.11), and (C_38_H_34_N_8_O_8_S_4_Zn, 922.07) respectively.

### Thermal analyses

The thermal analyses of synthesized compounds are recorded, utilizing thermogravimetric analysis (TGA), derivative thermogravimetric analysis (DTG), and differential thermal analysis (DTA), Figures (S5-S11), and the thermal parameters are collected in Table (S1). The correlation between the different decomposition steps of the compounds with the corresponding % weight losses are proposed in Schemes (S8-S14). The (TGA) curve of HL, Figure (S5) showed five decomposition steps, and the residual percentage mass equivalent to 14.23% (calc. 15.73%) is attributed to the un-sublimated carbon (4 C). These decomposition processes displayed maximum DTG peaks at 101.25 ^o^C, 276.76 ^o^C, 524.24 ^o^C, 626.63 ^o^C, and 706.48 ^o^C, accompanied with two endothermic DTA peaks at 263.07 and 512.73 ^o^C. Scheme (S8) represents the proposed mechanisms to TGA mass losses of HL. The TGA and DTG of HL^1^, Figure (S6), showed that the decomposition of HL^1^ happened in two stages, ended by a mass loss which may correspond to the remaining C_4_H_4_ residue. These decomposition steps are accompanied by two well characterized DTG peaks at 439.26 °C, and 657.48 °C. The DTA curve shows one exothermic peak at 36.87 °C, and two endothermic peaks at 362.41 °C and 524.22 °C. The thermal decomposition of HL^2^, Figure (S7), occurs in two main steps. The decomposition is continuous till 800 °C. The residual weight loss is equivalent to C_4_H_2_ residue Scheme (S9). These two steps are accompanied by two peaks of DTG at 212.36 °C and 587.18 °C and accompanied with three endothermic peaks at 357.35 °C, 417.06 °C, and 524.22 °C consequently. The TGA/DTA of metal complexes are illustrated in Figures (S8-S11). The (TGA) curve of [Cu(L^1^)₂]0.5 H₂O, showed six decomposition steps. The first step in the temperature range (45.76–147.66 °C) showing a weight loss of 1.862% corresponding to hydration water molecules calculated as 1.90% in the proposed structure, and the residual percentage mass equivalent to 13.012% (calc. 12.50%) is attributed to the un-sublimated CuO + C_3_. Scheme (S11) represents the proposed mechanisms to TGA mass losses of [Cu(L^2^)₂]0.5 H₂O. These decomposition processes display a maximum DTG peaks at 93.42^o^C, 287.55^o^C, 418.84^o^C, 528.00^o^C, 670.36 ^o^C and 792.85^o^C. The DTA curve showed one exothermic peak at 114.81 °C, and two endothermic peaks at 206.54 °C and 515.08 °C. The (TGA/ DTG) profile of the corresponding copper complex [Cu(L^2^)₂]0.3 H₂O proceed in two degradation steps through a temperature ranges; 43.24–393.43 °C, and 394.42–797.86 °C with mass loss 28.353% (calc.28.33%) and 52.126% (52.61%) consequently leaving CuS + C_7_ as residue with mass 19.521% (calc. 19.04%). The mechanism of decomposition is illustrated in Scheme (S12). These two steps are accompanied with two peaks of DTG at 230.93 °C and 566.76 °C, accompanied by two exothermic DTA peaks at 70.3 °C and 131.53 °C followed by endothermic DTA peak at 274.78 °C. The [Zn(L^1^)₂].2H₂O, exhibited the following thermal decomposition steps: The first step at 27.19–153.70 °C temperature range shows a weight loss of 4.229% which agrees with the calculated value of 4.06%, corresponding to non-coordinated water molecules. In the temperature range of 154.73–400.68 °C with the weight loss of 34.679% (calculated value is 35.05%). The weight loss of 42.074% compared with the theoretical value 42.03% in the 400.68–603.18 °C temperature range. The last step is assumed to be occurred in the temperature range of 604.15–798.70 °C with the mass loss of 5.254% compared with the calculated value of 4.94%. The TGA curve showed a residual 13.667% of the mass 13.93% is the calculated value, corresponding to Zn and C_5_ as residue, Scheme (S13). These four steps are accompanied with four peaks of DTG at 101.61 °C, 275.78 °C, 502.34 °C, and 646.12 °C. The DTA curve illustrated two exothermic peaks at 204.57 °C and 298.65 °C. The TGA/DTG curves of [Zn(L^2^)₂].2H₂O complex represent four decomposition steps within the temperature ranges of 47.47–224.75 °C, 225.72–480.81 °C, 481.55–668.03 °C, and 668.66–997.51 °C and the mass losses 4.759% (calc. 4.12%), 27.069% (calc. 27.11%), 30.622% (calc. 30.81%), and 21.006% (calc. 21.04%). The degradation steps ended by a mass loss of 16.544% (calc. 16.90%) which may correspond to the remaining ZnS + C_5_ residue. The thermal pattern of decomposition is out lined in Scheme (S14). These decomposition steps are accompanied with four DTG peaks at 134.98 °C, 330.78 °C, 590.46 °C, and 747.31 °C. The DTA curve Figure (3.47), is given one exothermic peak at 90.44 °C and one endothermic peak at 183.21 °C.

Furthermore, as the temperature increases, a series of thermal changes can be observed in the differential thermal analysis (DTA) curves of the free ligands and their copper (II) and zinc (II) complexes that were produced, Table (S3). The computed collision number (Z) of the complexes is directly related to (Ea). The minimum and maximum (Z) values are 44.821 and 776.484, respectively, indicating distinct mechanisms with varying speeds. The computed (Ea) values range from 44.431 to 580.89 JK^− 1^mol. The high (Ea) values represent the complexes’ thermal stability and suggest that the processes comprise translational, rotational, and vibrational states, as well as changes in mechanical potential energy for complexes. The decomposed substance fraction values (α_m_) at the maximal development of the reaction in each stage are almost the same magnitude, ranging from 0.444 to 0.651. The DTA endothermic and exothermic peaks have minimum and maximum T_m_ values of 309.87 and 797.57, respectively. The entropy values (ΔS) for free ligands and their complexes are essentially identical, ranging from − 0.189 to 0.218 kJ K^− 1^mol^− 1^. The negative sign indicates that the transition states have more ordered and less random chemical configurations than the reacting complexes, and/or that the reactions are sluggish [81]. The fractional values of the reaction order (n) Table (S3) indicate incomplete reactions or intricate mechanisms [82]. Negative (ΔH) values indicate exothermic breakdown procedures. The Arrhenius plots of thermal decomposition steps had a correlation coefficient of 0.992–0.999, indicating a satisfactory fit with a linear function.

### Dyeing properties of free ligands and their complexes

#### Dyeing and fastness properties

The dyeing color of the substrate was expressed using CIELAB coordinates, which included measurements of lightness (L), (a) (red-green axis), (b) (yellow-blue axis), chroma (c), and hue angle ranging from 0 to 360° (h). The positive values of (b), namely 18.34, 32.99, 24.78, 39.84, 27.87, and 32.29, indicate that the hues of dispersion dyes 1–3 and 7–9 on the substrate shifted towards a yellowish trend [83]. The disperse dye 1(HL) exhibited a shift towards a greenish color on the substrate, as indicated by the negative value of (a) = -1.78.

#### Fastness properties

Washing, Rubbing and Perspiration fastness. The washing fastness of dyed fabrics for all dyes under investigations have excellent washing fastness, as shown in Table 1. The results of the acquired data outline indicate that the colored substrate has extremely good fastness for rubbing and perspiration. The excellent intra-fiber diffusion of the dye molecules inside the substrate, along with the expected relatively large dye molecule particle size, may be the reason for these results [84, 85].

**Table 1:** Fastness properties of dye compounds on polyester

Dye Number	Washing on polyester		Perspiration on polyester		Rubbing on polyester	
	(St.)	(Alt.)	Acid	Alkaline	Dry	Wet
Dye 1 HL	5	4–5	4–5	5	5	4–5
Dye 2 HL^1^	5	5	5	5	5	4
Dye 3 HL^2^	5	5	4–5	5	5	5
Dye 4 [Cu(L^1^)₂]0.5 H₂O	5	5	4	5	5	4–5
Dye 5 [Zn(L^1^)₂]0.2 H₂O	5	5	5	5	5	5
Dye 6 [Zn(L^2^)₂].2H₂O	5	5	3	5	5	5

St. = Staining, Alt. = Alteration

### Biological studies

#### In vitro antimicrobial activity

The synthesized compounds are tested for their inhibitory effects on the growth of two pathogenic Gram-positive bacterial strains *Staphylococcus aureus* and *Bacillus subtilis*, and two pathogenic Gram-negative bacterial strains *Escherichia coli* and *Proteus vulgaris*, and two pathogenic fungi *Aspergillus flavus* and *Candida albicans*. Gentamicin served as standard for bacteria, and Ketoconazole served as standard for fungi. The antibacterial and antifungal activities of the new compounds are presented in Table 2. The antimicrobial screening data revealed that the investigated compounds possess antimicrobial properties against most of the tested organisms. However, HL is found to have relatively high activity against *Aspergillus flavus* but has no activity against *Candida albicans* while has activity against the gram positive and negative bacteria. HL^1^ is found to be active against the gram positive and negative bacteria but inactive against fungi. HL^2^ is found to be of better activity towards the studied pathogens and even showed better activity than the Ketoconazole drug itself. From the synthesized metal complexes, copper(II) complexes are found to be active against *Aspergillus flavus.* Complexes have activity against gram positive bacteria but have no activity against *Bacillus subtilis.* For the gram-negative bacteria, both [Cu(L^1^)₂]0.5 H₂O, and [Cu(L^2^)₂]0.3 H₂O, are found to be active, whereas [Cu(L^2^)₂]0.3 H₂O showed no activity against *Proteus vulgaris*. Similar to other studies, Cu(II) and Zn(II) complexes showed higher antibacterial activities [86, 87]. It worth to mention that the pathogens are disposed to inactivation by species that facilitate their diffusion through the lipid layer of the spore membrane to the site of action and kill them by combining with the OH and C = N groups of certain cell enzymes. The variation in the activity of the metal complexes against different organisms depends on the impermeability of the microorganism cells or on differences in ribosome of microbial cells. A number of factors, including the geometrical structure of the complexes, the nature of the donor atoms and metal ions, and the chelation of the ligands, can influence the complexes’ biological activity by increasing the molecule’s lipo-solubility and facilitating its passage through the bacterial membrane’s lipid bilayer [86, 88–91]. Nevertheless, the chelation process decreases the polarity of the metal ion due to the partial sharing of its positive charge with the donor groups and the potential π-electron delocalization within the chelate ring system that forms upon coordination [92, 93]. This boosts the rate of uptake/entrance and consequently enhances the antibacterial activity of the testing compounds. Thus, the complexes’ antimicrobial activity can be attributed to their enhanced lipophilic nature, which inactivates enzymes involved in respiratory processes and potentially other cellular enzymes crucial for the metabolic pathways of the bacteria being tested.

**Table 2:** The Inhibition diameter zone values for synthesized compounds

Sample code	Gram Positive Bacteria		Gram Negative Bacteria		Fungi	
	*Staphylococcus aureus*	*Bacillus subtilis*	*Escherichia coli*	*Proteus vulgaris*	*Aspergillus flavus*	*Candida albinos*
Standard*	24 ± 0.15	26 ± 0.41	30 ± 0.15	25 ± 0.32	16 ± 0.22	20 ± 0.32
Control: DMSO	Nil	Nil	Nil	Nil	Nil	Nil
HL	14 ± 0.29	12 ± 0.23	13 ± 0.19	12 ± 0.22	15 ± 0.31	Nil
HL^1^	13 ± 0.25	11 ± 0.13	10 ± 0.11	11 ± 0.23	Nil	Nil
[Cu(L^1^)₂]0.5 H₂O	12 ± 0.14	11 ± 0.08	8 ± 0.13	9 ± 0.10	8 ± 0.04	Nil
[Zn(L^1^)₂]0.2 H₂O	12 ± 0.44	Nil	11 ± 0.14	12 ± 0.61	Nil	Nil
HL^2^	12 ± 0.17	13 ± 0.04	12 ± 0.41	10 ± 0.34	20 ± 0.44	10 ± 0.29
[Cu(L^2^)₂]0.3 H₂O	8 ± 0.05	Nil	Nil	Nil	9 ± 0.22	Nil
[Zn(L^2^)₂].2H₂O	10 ± 0.25	13 ± 0.23	10 ± 0.28	9 ± 0.64	Nil	Nil

Standard*: Gram Positive and negative Bacteria; Gentamycin, fungi; Ketoconazole

#### In vitro cytotoxicity screening

Cytotoxicity is one of the most important indicators for biological evaluation in vitro studies. In vitro, chemicals have different cytotoxicity mechanisms such as obliteration of cell membranes, inhibition of protein synthesis or irreversible binding to receptors. While the focus of cancer treatment research is on creating new metal complexes similar to cisplatin, there is a rising interest in non-platinum metal complexes that have shown promising anticancer properties [94].

Conducting an initial study on the anticancer activity of the synthesized free ligands and their complexes, we evaluated their effectiveness against a specific human cell line, Lung carcinoma (A-549). Various doses of synthesized compounds were utilized to calculate IC_50_ values (the concentration required to inhibit 50% of culture development when cells were exposed to the tested compounds for 48 h), with vinblastine sulfate serving as a reference. The screening results are presented in Table 3. Based on the data obtained, it was found that the human tumor cell line showed different levels of sensitivity to the compounds that were tested. Among these, the human Lung carcinoma A-549 cell line showed pronounced sensitivity against HL^2^, and [Zn(L^2^)₂].2H₂O with IC_50_ values of 7.43, and 9.8 𝜇g/mL, respectively. In addition, a significant to moderate cytotoxic activity was displayed by compounds [Cu(L^1^)₂]0.5 H₂O and [Cu(L^2^)₂]0.3 H₂O giving 14.6 and 19.9 𝜇g/mL, respectively. Compound [Zn(L^1^)₂].2H₂O was able to exhibit mild activity against the same cell line with IC_50_ value 61 𝜇g/mL. Poor cytotoxic activities were displayed by HL^1^, HL, at IC_50_ values 106 and 143 𝜇g/mL, respectively. These findings illustrate how variations in the molecular and electron structures of chelation can lead to notable differences in the anticancer activity [94].

**Table 3:** IC_50_ values (µg/ml) of free ligands and their complexes and vinblastine sulfate standard drug in lung carcinoma (A-549)

Test compounds	IC_50_ (µg/mL)
	A-549 Lung cancer
Vinblastine Sulfate	24.6 ± 0.7
HL	143 ± 4.9
HL^1^	106 ± 6.1
[Cu(L^1^)₂]0.5 H₂O	14.6 ± 0.9
[Zn(L^1^)₂]0.2 H₂O	61 ± 2.3
HL^2^	7.43 ± 0.4
[Cu(L^2^)₂]0.3 H₂O	19.9 ± 1.3
[Zn(L^2^)₂].2H₂O	9.8 ± 0.9

#### Antioxidant activity (DPPH radical scavenging activity)

The antioxidant activity of the substances is assessed in vitro using the DPPH (diphenyl picryl hydrazyl) free radical scavenging activity, which is measured in terms of IC_50_ (the concentration in µg/mL required to block DPPH radical production by 50%) [95], Table 4. Among the free ligands, the HL^1^ showed lowest IC_50_, displaying good reducing ability of DPPH radical. The ligands, on the other hand, are more effective scavengers than their complexes. One possible explanation is that HL^1^ contains free amino and hydroxyl groups [96] and free amino and thiol groups in HL^2^. The ability to reducing of DPPH radical of free ligands decreases in the sequences: HL^1^ > HL > HL^2^. The tested HL^1^ complexes compared to standard Ascorbic acid as [Cu(L^1^)₂]0.5 H₂O has higher efficiency than [Zn(L^1^)₂].2H₂O while zinc complex of HL^2^, [Zn(L^2^)₂].2H₂O was found to have higher efficiency than copper(II) complex, [Cu(L^2^)₂]0.3 H₂O. This may be attributed to the nature of metal ion where Cu(II) is a d^9^ electronic configuration ion, while Zn(II) is a d^10^, with variable redox activities, in addition to the chelate effect of the ligands, where L^1^and L^2^ are different in chelating centers: L^1^(O and N), while in L^2^ (S and N). Both factors mainly affect the polarity of the metal ions, thus their redox behavior.

**Table 4:** IC_50_ values (µg/ml) of free ligands and their complexes and ascorbic acid as standard

Compounds	IC_50_ (µg/mL)
HL	55.8 ± 0.44
HL^1^	27.2 ± 0.90
[Cu(L^1^)₂]0.5 H₂O	110.9 ± 0.24
[Zn(L^1^)₂]0.2 H₂O	207.5 ± 0.13
HL^2^	107.3 ± 0.84
[Cu(L^2^)₂]0.3 H₂O	347 ± 0.37
[Zn(L^2^)₂].2H₂O	300.5 ± 0.16
Ascorbic acid	14.2 ± 0.27

### Computational study

In this section of the study, we are expressing [Cu(L^1^)₂]0.5 H₂O as Cu1, [Cu(L^2^)₂]0.3 H₂O, Cu1, [Zn(L^1^)₂].2H₂O as Zn1 and [Zn(L^2^)₂].2H₂O as Zn2, for simplicity.

#### Geometrical optimization

**Fig. 7:**
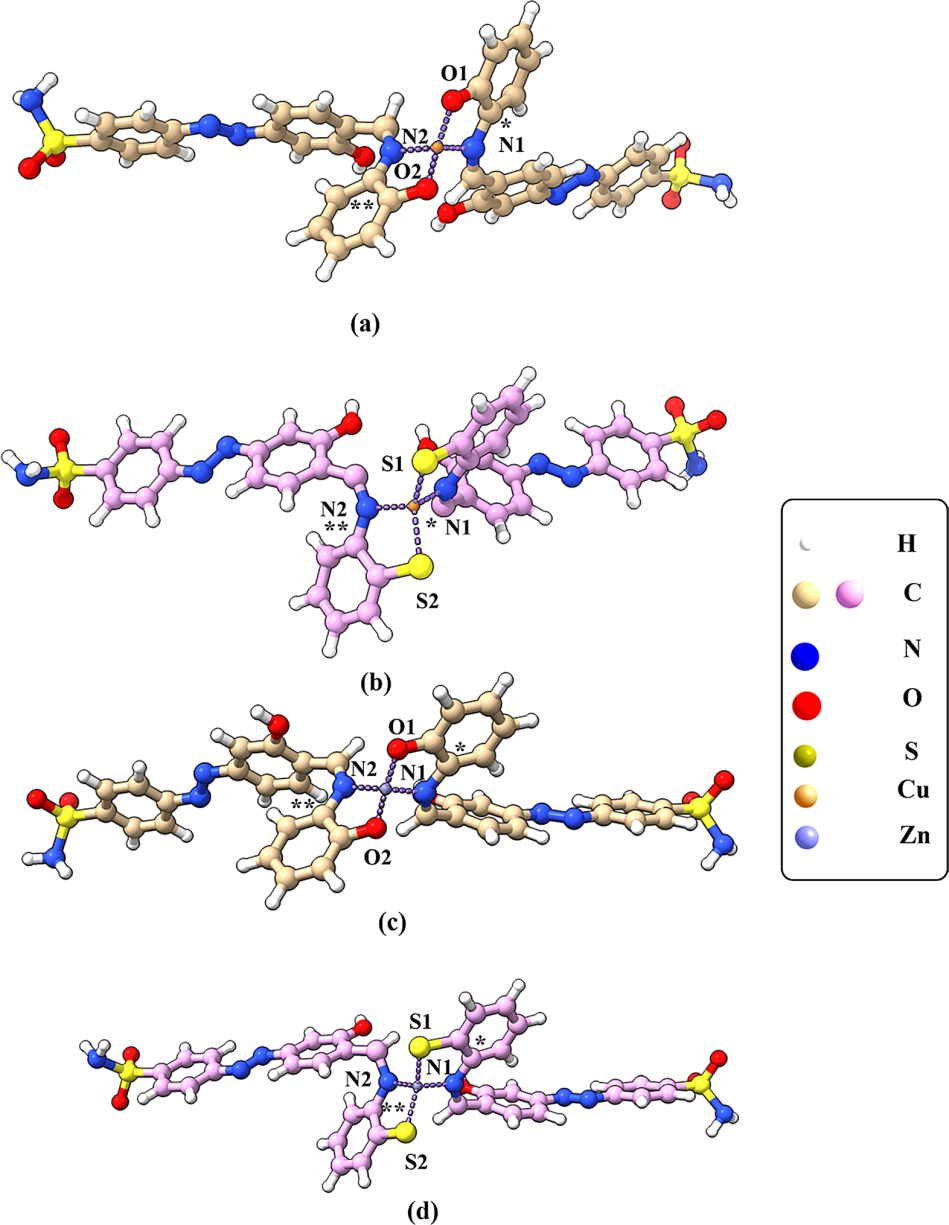
The optimized structures with DFT/LANL2DZ method, for (**a**) Cu1 and (**b**) Cu2, (**c**) Zn1 and (**d**) Zn2

Optimization of Cu(II) and Zn(II) complexes were achieved, and some important geometrical parameters were evaluated indicating the predicted distorted geometrical structures. Figure 7 shows the optimized metal complexes coordinated with 2 oxygen atoms and 2 nitrogen atoms for Cu1 and Zn1, while the other studied complexes are coordinated with 2 sulfur atoms and 2 nitrogen atoms for both Cu2 and Zn2. The applied geometrical parameters for illustration of the structure of copper and zinc complexes are shown in Table 5. The calculated results from DFT showed that Cu1 is found in distorted tetrahedral geometrical structure, with a dihedral angle − 6.76⁰ and − 0.44⁰ for O1CuN1C* and O2CuN2C** planes, this finding describes the presence of O1 donor atom in vertical axis with Cu coordinated center, while O2 donor atom occurs in perpendicular with Cu coordinated center. Consequently, the bond lengths in this complex show a slight interaction enhancing in M-O1 and M-N1 (1.820Å and 1.849Å, respectively). The bond angles of N1-Cu-N2, N1-Cu-O2, N2-Cu-O1 and O1-Cu-O2 are 104.3⁰, 124.2⁰, 120.5⁰ and 120.1⁰, respectively, which confirm the distorted tetrahedral structure. Furthermore, Cu2 appears to be a distorted tetrahedral environment, with dihedral angles, -10.11⁰ and 6.41⁰ for S1CuN1C* and S2CuN2C** planes. The results revealed that N1 and S1 donor atoms with a slight less interaction than the corresponding and this also, based on the bond lengths displayed. Additionally, the bond angles investigated the distorted geometrical optimized structure. For Zn1, its calculated geometrical parameters revealed the structure in distorted tetrahedral environment, with dihedral angles; 6.02⁰ and 1.07⁰ for O1ZnN1C* and O2ZnN2C** planes, respectively. The bond lengths and dihedral angles confirmed the findings where O2 and N2 donor atoms more strongly interacted with the metal center locating at the same plane in distorted tetrahedral structure. As the same, Zn2 shows a distorted tetrahedral geometrical structure, with a dihedral angle − 12.14⁰ and − 0.081⁰ for S1CuN1C* and S2CuN2C** planes, respectively. The results also confirm the strong interaction of S2 and N2 atoms with Zn atom in the same plane distorted coordinated structure.

**Table 5:** Some important geometrical parameters (bond length and bond angle of the optimized copper and zinc complexes

Atom number	Cu1	Cu2	Zn1	Zn2
Bond length (Å)				
M-N1	1.849	1.871	1.793	1.777
M-N2	1.841	1.869	1.785	1.802
M-O1	1.820	------	1.738	------
M-O2	1.813	-------	1.747	-------
M-S1	-----	2.253	------	2.171
M-S2	-----	2.246	------	2.158
**Bond angle (** ^**o**^ **)**				
N1-M-N2	104.3	105.730	113.426	108.498
N1-M-O1	96.3	-------	99.655	------
N1-M-O2	124.2	-------	116.418	-------
N1-M-S1	------	100.487	-------	101.514
N1-M-S2	------	119.407	--------	113.942
N2-M-O1	120.5	-------	116.502	-----
N2-M-O2	92.3	-------	99.138	------
N2-M-S1	------	117.665	------	116.143
N2-M-S2	------	98.205	------	101.986
O1-M-O2	120.1	-------	112.623	------
S1-M-S2	------	115.923	-------	115.082

**Fig. 8:**
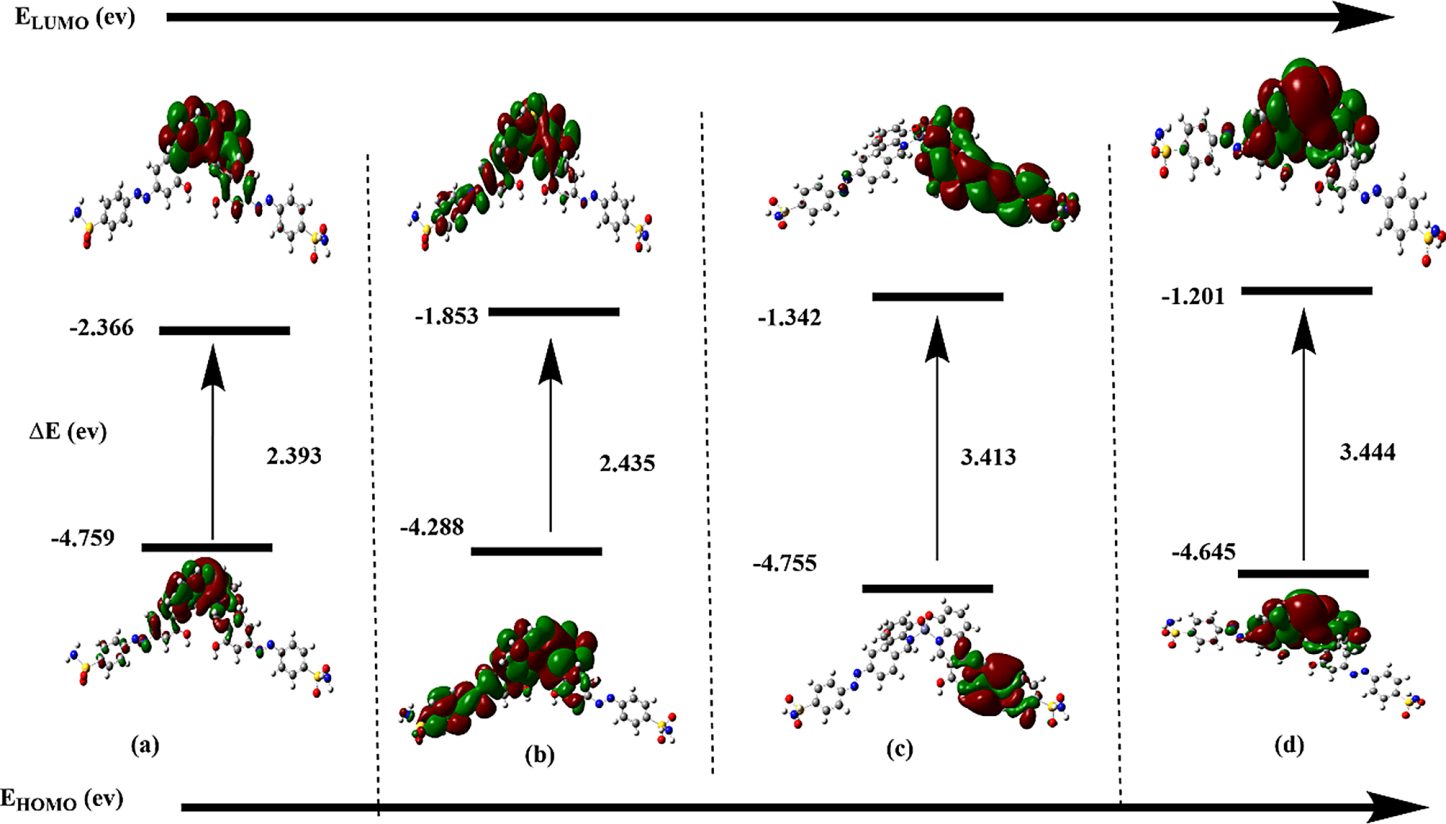
FMOs energies calculated by DFT/LANL2DZ method in gas phase for (**a**) Cu1 and (**b**) Cu2, (**c**) Zn1 and (**d**) Zn2

In more detailed structural investigation, the FMOs analysis was considered to identify the orbital contribution in the studied complexes supported with molecular orbital energies describing the stability conditions of the structure. Figure 8 shows the FMOs map, where the energy gap (∆E = E_HOMO_-E_LUMO_) mainly controls the electronic transition. According to the supported results, Cu complexes exhibit ease in electronic transitions as ∆E values of Cu1 and Cu2 are 2.393 eV and 2.435 eV, respectively. while the more stable Zn complexes exported higher in ∆E values, 3.413 eV and 3.444 eV for Zn1 and Zn2, respectively. it was noticed that the HOMO and LUMO contribution located around the coordination area of donor and acceptor sites and this may be an indication about stability index for complex formation.

#### Molecular electrostatic potential (MEP)

**Fig. 9:**
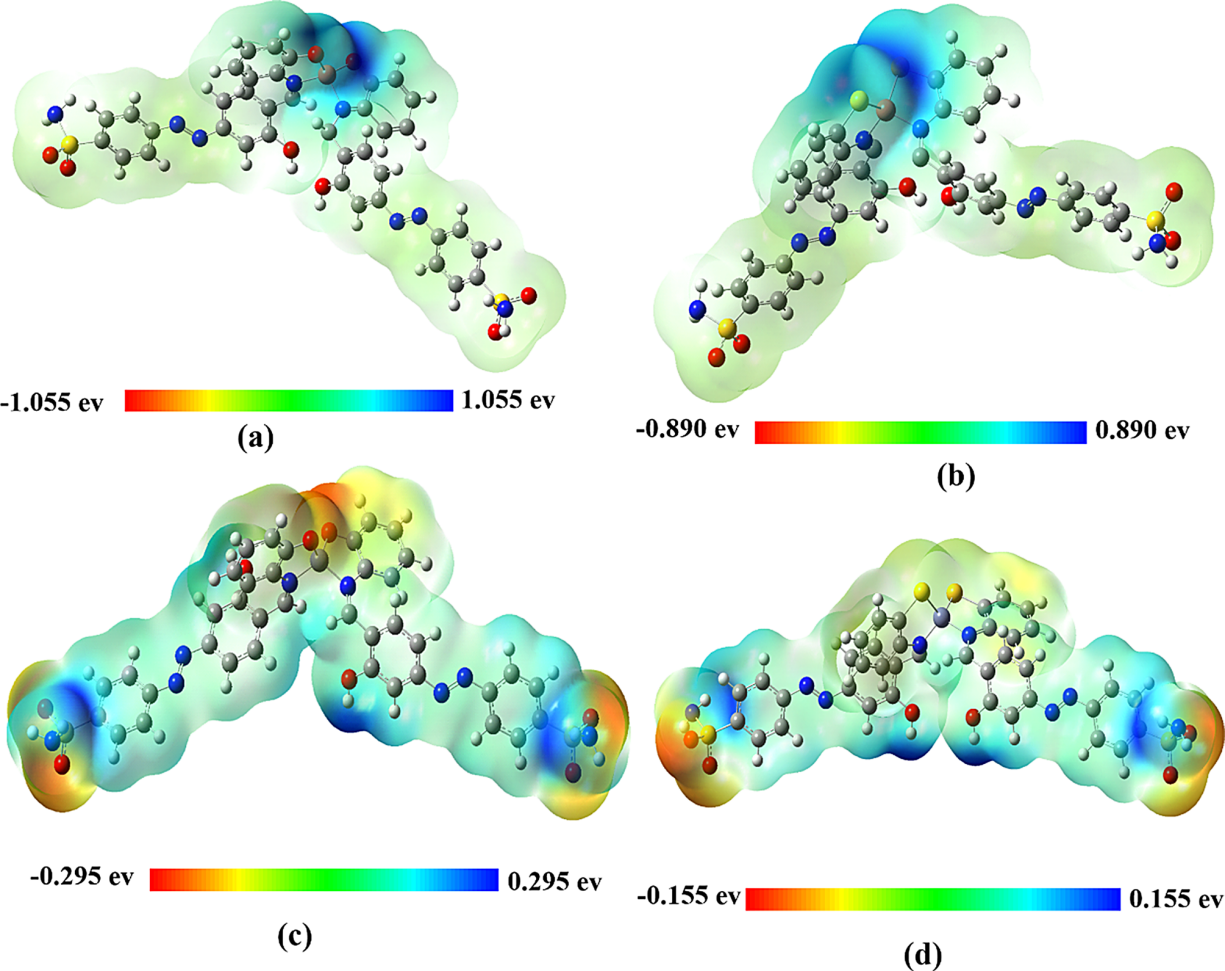
3D-MEP surface for (**a**) Cu1 and (**b**) Cu2, (**c**) Zn1 and (**d**) Zn2

MEP stands for Molecular Electrostatic Potential, which is a representation of the electrostatic potential on the surface of a molecule with a constant electron density. It is mostly used in investigations related to biological recognition and H-bond interactions to predict the relative reactivity of molecular sites towards electrophilic processes [97]. The mapped MEP scheme for the optimized structures under study is depicted in Fig. 9. The blue regions indicate areas of lesser electron density, which correlate to positive electrostatic surface potential (ESP). Conversely, the red regions indicate areas of higher electron density, which correspond to negative ESP. The results elucidated the existence of active areas within the coordinating environment. In case of Cu complexes, the oxygen and sulfur donor atoms coordinated with high less electronic density than in Zn complexes. This may be attributed to the type of metal center as the interacting metal orbitals differ in electronic contribution. In more details, the unpaired electron in 3d-orbital of Cu atom can increase the strength of interaction with ligand donor atoms, while the electrons-filled in 3d orbital for Zn atom may decrease the interaction with the ligand donor atoms leading to electronic localization on the Oxygen and Sulfur surround Zn center.

#### Infra-red spectra

**Fig. 10:**
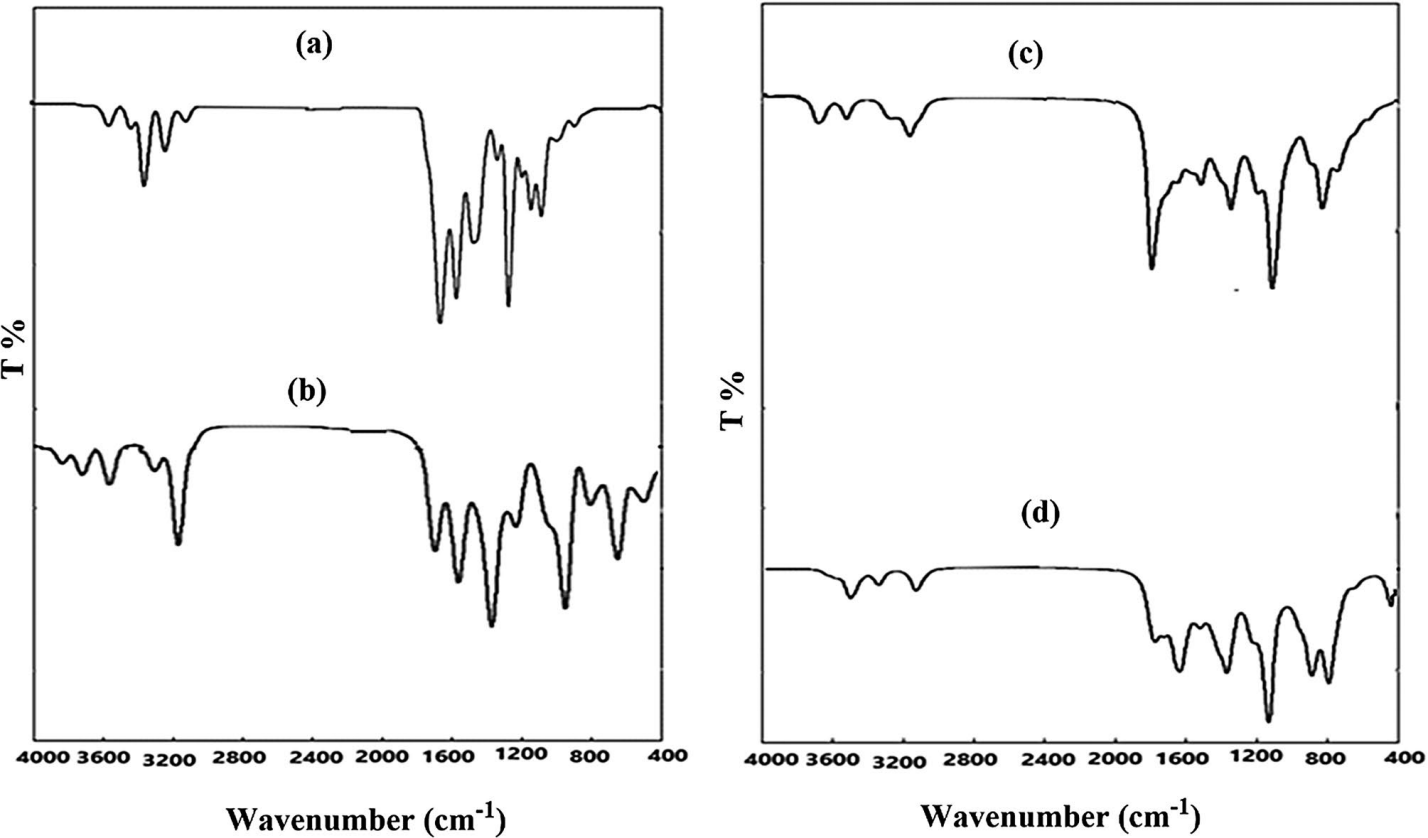
Calculated IR using DFT/LANL2DZ method in gas phase for (**a**) Cu1, (**b**) Zn1, (**c**) Cu2 and (**d**) Zn2

As shown from Figs. 10(a & b), the IR spectra of optimized Cu1 and Zn1 agree with the experimental results as there is a band at 1600 cm^− 1^ that corresponds to the azomethine-nitrogen (C=N) that bonded to the metal ion (Cu or Zn). We show a band at 1260 cm^− 1^ which corresponds to the coordinated C-O phenolic group of aminophenol in both complexes. The characteristic band of non-coordinated C-O phenolic group appears as a small, interfered band at 1318 cm^− 1^ in Cu complex but in zinc complex, it appears as a small separate band at 1327 cm^− 1^. The band at about1470 cm^− 1^ corresponds to the non-bonded -N=N- group to the metal. In the case of IR of Cu2 and Zn2, Figs. 10(c & d), the band of S-H that the band experimentally disappeared, and the theoretical results confirmed this. According to the experimental data, a small and relatively broad band appears at 1590 cm^− 1^, corresponding to the C=N group, which was expected to be coordinated with the metal. Additionally, the non-bonded -N=N- group to the metal is related to the band at around 1470 cm^− 1^.

#### Electronic explanation using TD-DFT theory (UV– Vis spectra)

**Fig. 11:**
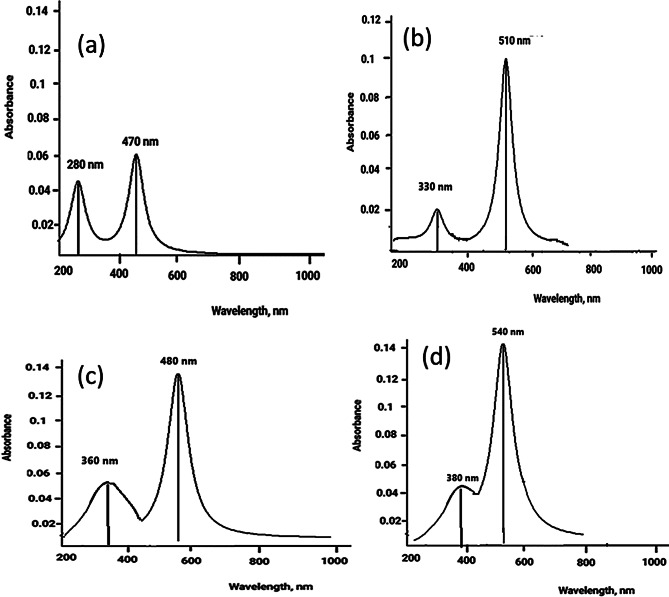
UV-Vis electronic absorption using TD-DFT/B3LYP method and LANL2DZ basis set for (**a**) Cu1, (**b**) Cu2, (**c**) Zn1 (**d**) Zn2

To study the electronic absorption spectra, TD-DFT/B3LYP at LANL2DZ method is applied on the optimized complex structures in ground state, considering PCM model, in ethanol. Cu complex I, Fig. 11(a), shows two electronic absorption maxima: at 470 (f = 0.018, E = 1.601 eV) corresponding to π- π* transition for 92.3% contribution of HOMO-1→LUMO and 23.1% contribution of HOMO-1→HOMO transitions. The second transition corresponding to wavelength of 280 nm (f = 0.037, E = 2.088 eV) represents n-π* transition of 70.9% contribution of HOMO→LUMO + 2 and 16.8% contribution of HOMO → LUMO transition.

In Fig. 11(b), Cu2 shows two electronic absorption spectra at 510 nm (f = 0.051, E = 1.913 eV) corresponding to π- π* transition for 70.4% contribution of HOMO→LUMO + 1 and 44.6% contribution of HOMO-1→LUMO transitions and, the other at 320 nm (f = 0.0902, E = 2.285 eV) corresponding to two contributions for 67.6% contribution of HOMO→LUMO and 50.5% LUMO→LUMO + 1.

Applying TD-DFT analysis on Zn1, it is shown from Fig. 11(c), that there are two absorption bands, one at wavelength of 480 nm (f = 0.0378, E = 0.4281ev) Where the transition is predicted to be from HOMO to LUMO, corresponding to n-π* transition for 96.5% contribution. The other band at 360 nm (f = 0.0156, E = 0.935 eV). The other band at 360 nm, this transition is contributed to be from HOMO to LUMO + 1 with 66.9% and from HOMO-2 to LUMO with 18.9%. Also, in the case of Zn2, Fig. 11(d) shows two transition bands, one at 540 nm (f = 0.069, E = 0.3646ev), where the transition is predicted to be from HOMO-1 to LUMO which corresponds to π-π* transition for 73.1% contribution. The other band at 380 nm (f = 0.036, E = 0.742 ev) from HOMO to LUMO (n- π transition) with orbital contribution 56.1%.

**Fig. 12:**
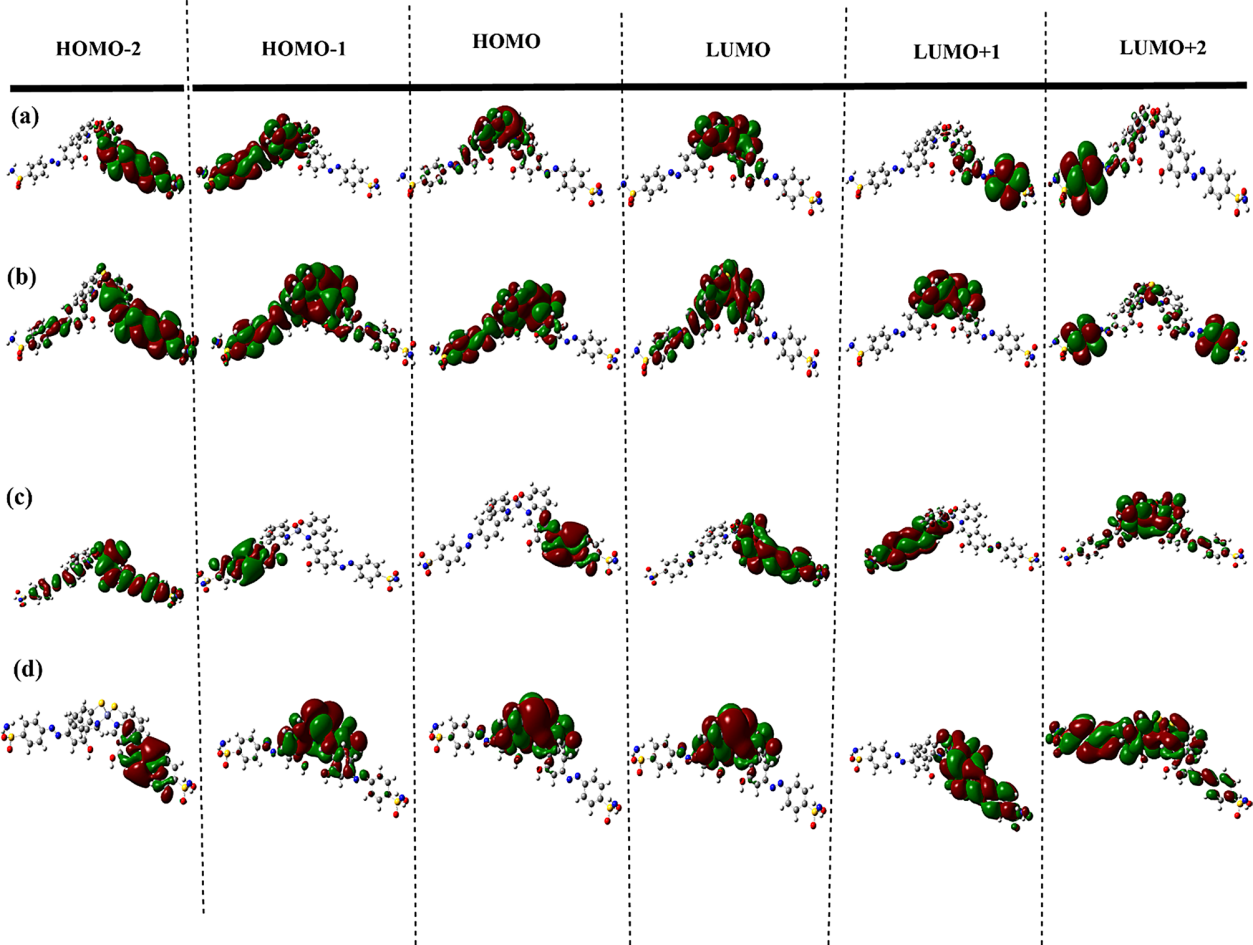
Electronic transition states with orbital contribution for (**a**) Cu1, (**b**) Cu2, (**c**) Zn1 and (**d**) Zn2

The results demonstrate a commendable alignment with the experimental data. Figure 12 show doublet transitions of molecular orbitals for Cu1, Cu2, Zn1, and Zn2 respectively, using TD-DFT(LANL2DZ). In Cu1 and Zn1 complexes, the orbital contribution on the metal center appears in only HOMO and LUMO, but in case of Cu2 and Zn2 complexes, the orbital contribution on the metal center appears on HOMO, HOMO-1, HOMO-2, LUMO, LUMO + 1 and LUMO + 2. This observation confirmed the higher stability of metals coordinated with sulfur other than oxygen-coordinated complexes.

#### Molecular docking analysis

Docking of the four complexes with S.aureus and *E-coli* give a good results in efficient inhibition of some sites of the DNA with formation of H-bond between the complex heteroatoms and amino acids of the receptor.

**Fig. 13:**
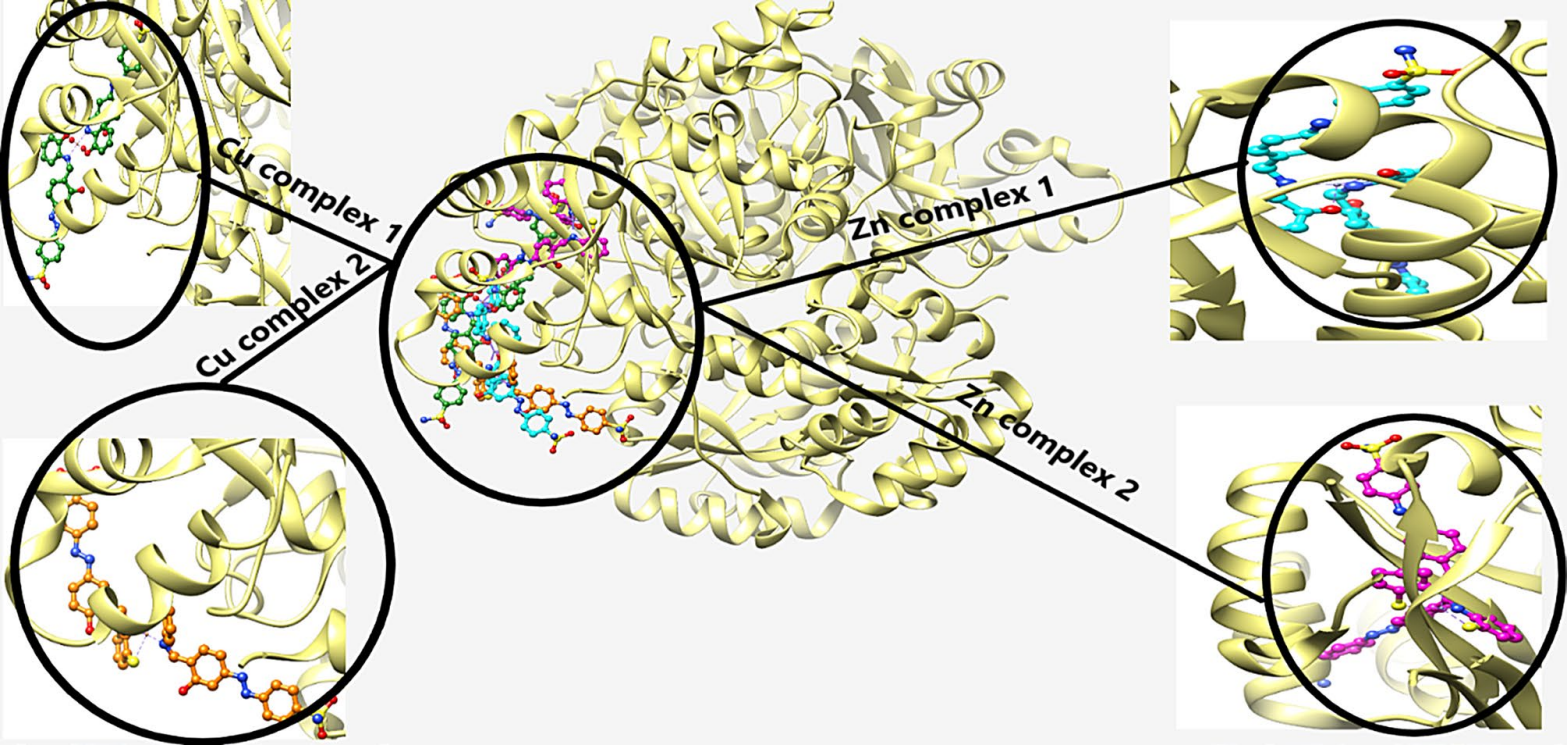
Molecular docking of the four studied complexes with *S. aureus* (5ND9)

**Fig. 14:**
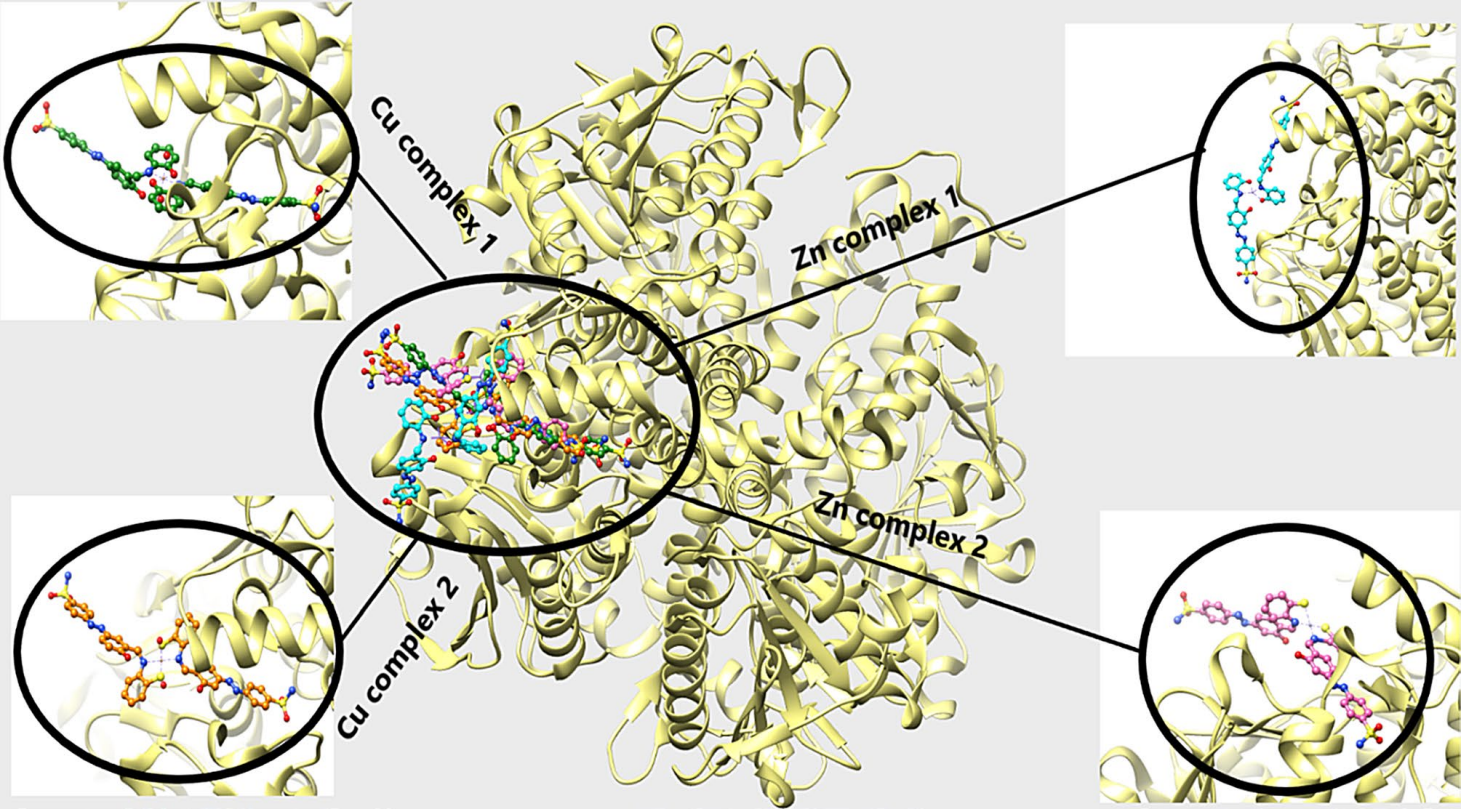
Molecular docking of the four studied complexes with *E-coli* (6QF6)

**Fig. 15:**
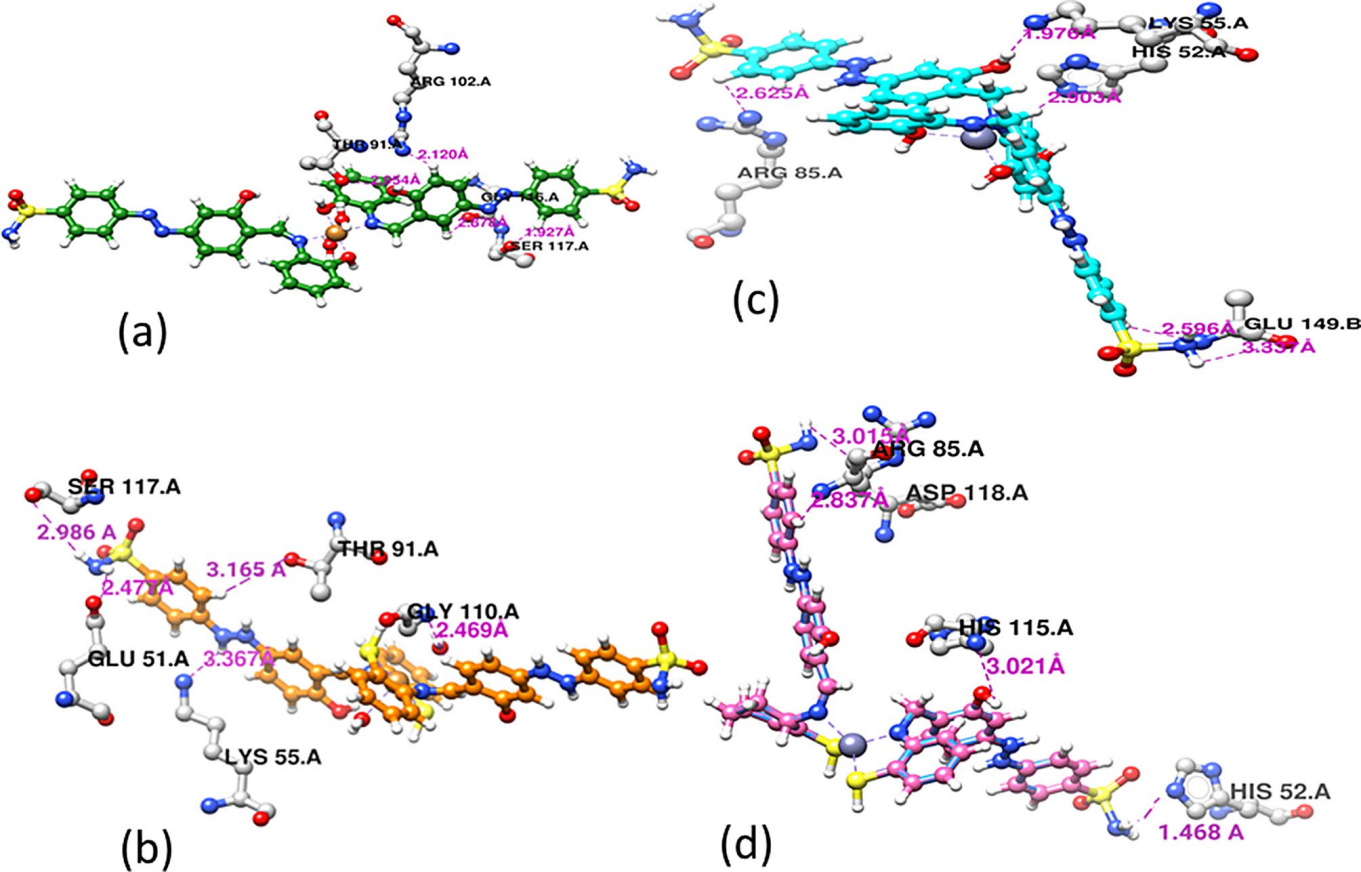
Hydrogen bond interactions with *S. aureus* for (**a**) Cu1 (**b**) Cu2 (**c**) Zn1 (**d**) Zn2

Figures 13 and 14 show the docking poses of complexes with S. aureus (5ND9) and E-coli (6QF6) targets where the four complexes appear to be closely docked in the same active site of the target. In Fig. 13, the docking energy of Cu1 is -89.5 kcal/mol and its interaction with different amino acids through H-bond formation is shown in Fig. 15(a). It is interacted with four amino acids namely ARG 102.A, THR 91.A, GLY 116.A and SER 117.A with H-bond lengths 2.120 Å, 2.954 Å, 2.878 Å and 1.927 Å, respectively.In case of Cu2 is -77.5 kcal/mol and it is interacted with five different amino acids shown in Fig. 15(b) namely SER 117.A, THER 91.A, GLY 110.A, GLU 51.A AND LYS 55.A with bond lengths 2.986 Å, 3.165 Å, 2.469 Å,2.477 Å and 3.367 Å, respectively. For Zn1, its docking energy with the target is -92.7 kcal/mol and as shown from Fig. 15(c), It is interacted with four amino acids namely ARG 85.A, LYS 55.A, HIS 52.A and GLU 149.B with H-bond lengths 2.625 Å, 1.976 Å, 2.903 Å and 2.596 Å, respectively. The docking energy of Zn2 is -103.3 kcal/mol and as shown from Fig. 15(d) its interaction with the target through four different amino acids namely, ARG 85.A, ASP 118.A, HIS 115.A and HIS 52.A with H-bond lengths, 3.015 Å, 2.837 Å, 3.021 Å and 1.468 Å, respectively.

**Fig. 16:**
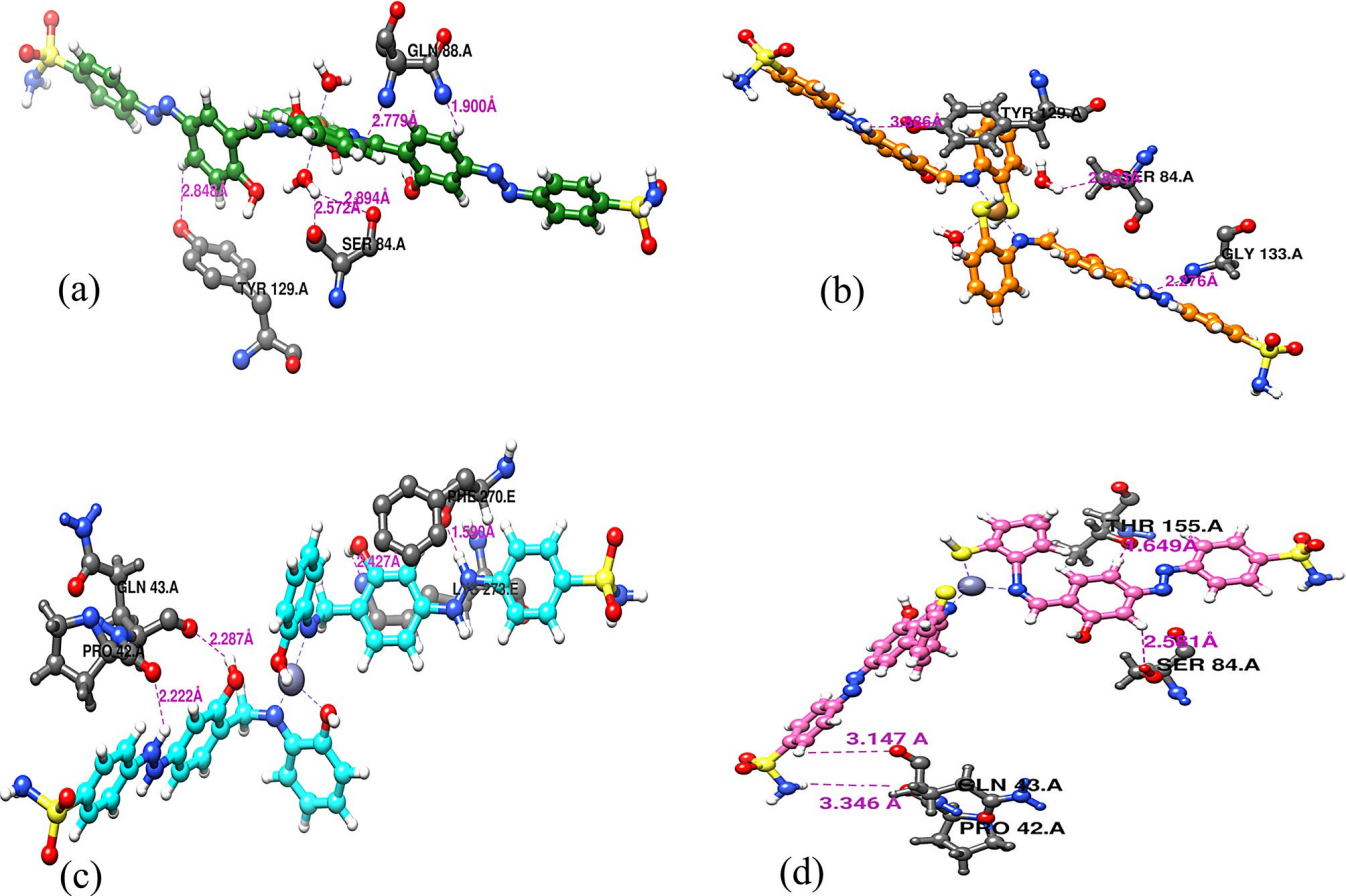
Hydrogen bond interactions with *E-coli* (6QF6) for (**a**) Cu1 (**b**) Cu2 (**c**) Zn1 (**d**) Zn2

With the same manner, the molecular docking of the four complexes with *E-coli* target is shown in Fig. 14 and their interaction with different amino acids through H-bond formation is shown in Fig. 16. The docking energies (fitness) for Cu1, Cu2, Zn1 and Zn2 are − 102.9 kcal/mol, -99.5 kcal/mol, -97.2 kcal/mol and − 109.3 kcal/mol, respectively.

## Conclusion

Two new azo-Schiff bases, HL^1^ and HL^2^ were synthesized and their interactions with copper(II) and zinc(II) salts were studied. Synthesis of azo compound HL, derived from sulfanilamide and salicylaldehyde was operated as starting step for the synthesis of the two new azo-Schiff bases, HL^1^ and HL^2^ via its condensation with 2-aminophenol and 2-aminothiophenol, respectively. The products were fully characterized utilizing different physicochemical techniques, such as UV-Vis, IR, NMR, mass spectra, thermal analysis (TGA, DTG and DTA), as well as EPR, molar conductivity measurements for metal complexes. The experimental data evidenced that the metals were ligated to the azo-Schiff bases, HL^1^ and HL^2^ via the azomethine-N and phenolic-O or thiolato-S, respectively. The compounds were applied as dyes for polyester fabric samples and their properties were studied. Their color fastness to washing, rubbing and perspiration were determined according to the ISO 105-C02:1989, ISO 105-X12:1987 and ISO 105-E04:1989 test methods, respectively. The dyed fabrics showed excellent washing fastness and very good fastness for rubbing and perspiration for all dyes under investigations which may attributed to their excellent intra-fiber diffusion inside the substrate. The synthesized metal complexes and their parent ligands were in vitro screened against panel of pathogenic bacterial strains; *Staphylococcus aureus* and *Bacillus subtilis* (Gram-positive bacteria), *Escherichia coli*, *proteus vulgaris*(Gram-negative bacteria) compared with Gentamycin and pathogenic fungi *Aspergillus flavus* and *Candida albicans*, compared with Ketoconazole. The cytotoxicity of synthesized compounds in one human cell line, Lung carcinoma (A-549) was investigated showing good example of how changes in the chelation and molecular structures could lead to insightful differences in anticancer activity. Furthermore, the antioxidant activities were studied, compared to the standard Ascorbic acid and their ability to reduce DPPH radical was also affected by the molecular composition and structure as for free ligands, the sequences of decreasing power was HL^1^ > HL > HL^2^ while, the attitude for metal complexes showed that [Cu(L^1^)₂]0.5 H₂O was found to be more powerful than [Zn(L^1^)₂].2H₂O whereas, [Zn(L^2^)₂].2H₂O >[Cu(L^2^)₂]0.3 H₂O. The applied computational studies and molecular docking on the proposed structures of the complexes were in good agreement with the experimental results.

## Electronic supplementary material

Below is the link to the electronic supplementary material.

Supplementary Material 1

## Data Availability

The authors declare that the data supporting the findings of this study are available within the paper. Should any raw data files be needed in another format they are available from the corresponding author upon reasonable request.

## Abbreviations

FT-IR: Fourier Transform Infra‑red spectra
NMR: Nuclear Magnetic Resonance spectra
EPR: Electron Paramagnetic Resonance spectra
MEP: Molecular Electrostatic behavior
DPPH: 2,2‑Diphenyl‑1‑picryl‑hydrazyl‑hydrate
DFT: Density Functional Theory
FMOs: Frontier Molecular Orbitals
HOMO: Highest occupied molecular orbital
LUMO: Lowest unoccupied molecular orbital
ELF: Electron localization function
MD: Molecular docking
