# Supplementary material for: Synthesis, characterization, computational and dyeing behavior of Cu(II) and Zn(II) metal complexes derived from azo-Schiff bases containing phenol derivatives
Source: BMC Chem. 2025 Jul 10;19(1):207. doi: 10.1186/s13065-025-01571-6 (PMC12243252; doi:10.1186/s13065-025-01571-6)
Supplement: Supplementary file 1 — Supplementary Material 1 [file 13065_2025_1571_MOESM1_ESM.docx]

**Synthesis, characterization, computational and dyeing behavior of Cu(II) and Zn(II) metal complexes derived from azo-Schiff bases containing phenol derivatives**

Hemmat A.Elbadawy^a,^^[[1]](#footnote-1)^ , Ali Eldissouky ^a^, Morsy Ahmed El-Apasery ^b^ , Doaa S. Elsayed ^a^, Entesar Ali Alsanosi Alaswad ^a^

^a^ Chemistry Department, Faculty of Science, Alexandria University, Egypt.
^b^ Dyeing, Printing and Textile Auxiliaries Department, Textile Research and Technology Institute, National Research Centre, Egypt

## **S.1. Materials and measurements**

Sulphanilamide (Qualikems Fine Chemicals Pvt. Ltd.), salicylaldehyde (Reg. off. Loba Chemie Pvt. Ltd.), 2-aminophenol (FSA Laboratory Supplies), 2-aminothiophenol , sodium dihydrogen orthophosphate dihydrate(NaH_2_PO_4_.2H_2_O), disodium hydrogen orthophosphate dihydrate (Na_2_HPO_4_.2H_2_O), and L-histidine monohydrochloride monohydrate, hydrochloric acid and nitric acid (Sigma-Aldrich Chemical Co.),sodium nitrite, sodium hydroxide, sodium bicarbonate, sodium chloride (El Nasr Pharmaceutical Chemicals Co.), Zinc(II) acetate dihydrate (BDO Chemicals Ltd.), copper(II) chloride dihydrate (Winlab laboratory Chemicals Reagents Chemicals), ethanol (DOP ORCANIK KIMYA SAN. VE TIC. LDT. STI.), spectroscopic grade dimethyl sulfoxide and dimethyl formamide (SDFCL s d fine-chem limited). All chemicals were used as received without further purification. CHN contents in each sample were determined using PerkinElmer, 2400 CHNS Elemental Analyzer 100V, carried out in temperature range from 100 - 1000°C. The metal content in each complex was determined complexometric using standard EDTA solution in presence of the suitable indicator and common methods[1]. Molar conductivity was determined by using HI8033 HANNA conductometer at 25 °C ± 1 for 1.00 x 10⁻^3^ M DMF solution. Melting points were measured on a Stuart Scientific melting point apparatus. The FT-IR spectra for all organic ligands and their metal complexes were recorded as KBr discs in the wave number (υ) 400-4000 cm⁻¹ range, utilizing a Perkin-Elmer Infrared Spectrophotometer (FT-IR 1650), at room temperature. The ¹HNMR and ^13^CNMR spectra of the new synthesized azo-Schiff bases and their Zn(II) complexes were recorded DELTA2_NMR500 MHz instrument as d⁶-DMSO solution in absence and in presence of D₂O using spectrometer in presence of tetra-methyl silane (TMS) as an internal standard, the figures in the text were presented using MestReNova software program. The electronic absorption spectra of for all compounds were recorded as DMF solution in the range of 200-1000 nm using automated spectrophotometer UV-Vis. Thermo Fischer Scientific Model Evolution 300 at room temperature. The room temperature X-band EPR spectra for polycrystalline copper (II) complexes were recorded using the X-band EPR spectrometer (Bruker, EMX) utilizing a high sensitivity standard cylindrical resonator (ER4119HS) operating at 9.8 GHz, with a 100 kHz modulation frequency, Modulation amplitude 1G, time constant 20.48 m sec, conversion time 81.92 m sec, microwave power 8 mW. Diphenyl picryl hydrazyl free radical (DPPH) was used as an internal reference. Electron impact mass spectrometry (EI-MS) of the compounds were recorded utilizing a Finnigan SSQ 7000 spectrometer attached to a digital DEC 300 workstation. Differential thermo gravimetric (DTG) analyses were carried out in the temperature range of 20 ̶ 1000 °C in a stream of nitrogen atmosphere by Shimadzu DTG 60H thermal analyzer. Thermogravimetric /Differential thermal analysis (TGA/DTA) analyses were carried out using SDT-Q600-V20.5-Build.

## **S.2. Biological Activity**

### **Method S1 *Invitro* antimicrobial activities**

The microbial activities of HL^1^, HL^2^ and HL^3^ and their complexes were screened against pathogenic bacterial strains *Staphylococcus aureus* and *Bacillus subtilis* as Gram-positive bacteria, *Escherichia coli, proteus vulgaris* as Gram-negative bacteria and pathogenic fungi *Aspergillus flavus* and *Candida albicans*. Antimicrobial activities of the tested samples were determined using a modified Kirby-Bauer disc diffusion method[2]. Stock solution of each sample was prepared by dissolving 5.0mg in 1mL of DMSO. Stock solutions were aseptically diluted two-fold to prepare solutions of different concentrations. The antibacterial and antifungal activities of the test compounds were assayed by filter paper disc method[3]. The activities were determined by measuring the diameter of the inhibition zone (mm) and media with DMSO was used as control. All cultures were routinely maintained on NA (nutrient agar) and incubated at 37 °C. The inoculums of bacteria were performed by growing the culture in nutrient agar broth at 37°C for overnight. Approximately, 0.1 mL of diluted bacterial or fungal culture suspension was spread uniformly on nutrient agar plates. Solutions of the test compounds and reference drugs were prepared by dissolving 10.0 mg of the compound in 10 mL DMSO. A 100 μL volume of each sample was pipetted into a hole (depth 3 mm) made in the center of the agar. Sterile 6 mm discs (Himedia Pvt. Ltd.) were impregnated with test compounds. The disc was placed onto the plate. Each plate had one control disc impregnated with solvent. The plates were incubated for 18-48 h at 37°C. Standard discs of Gentamycin (Antibacterial agents; 4.0 μg / ml) and Ketoconazole (Antifungal agent; 100.0 μg / m) served as positive controls for antimicrobial activity while filter discs impregnated with 10 μL of solvent DMSO were used as a negative control.

### ***Method S2 Invitro* cytotoxicity screening**

The mammalian cell lines: A-549 cells (human Lung cancer cell line), were obtained from VACSERA Tissue Culture Unit. Chemicals Used: Dimethyl sulfoxide (DMSO), crystal violet and trypan blue dye were purchased from Sigma (St. Louis, Mo., USA). Fetal Bovine serum, DMEM, RPMI-1640, HEPES buffer solution, L-glutamine, gentamycin and 0.25% Trypsin-EDTA were purchased from Lonza. Crystal violet stain (1%):is prepared from 0.5% (w/v) crystal violet and 50% methanol then diluted with distilled H_2_O, and filtered through a Whatmann filter paper (No.1).Cell line Propagation: The cells were propagated in Dulbecco’s modified Eagle’s medium (DMEM) supplemented with 10% heat-inactivated fetal bovine serum, 1% L-glutamine, HEPES buffer and 50µg/ml gentamycin. All cells were maintained at 37ºC in a humidified atmosphere with 5% CO_2_ and were sub-cultured two times a week. Cytotoxicity evaluation using viability assay: For cytotoxicity assay, the cells were seeded in 96-well plate at a cell concentration of 1×10^4^ cells per well in 100µl of growth medium. Fresh medium containing different concentrations of the test sample was added after 24 h of seeding. Serial two-fold dilutions of the tested chemical compound were added to confluent cell monolayers dispensed into 96-well, flat-bottomed microtiter plates (Falcon, NJ, USA) using a multichannel pipette. The microtiter plates were incubated at 37 ºC in a humidified incubator with 5% CO_2_ for a period of 24 h. Three wells were used for each concentration of the test samples. Control cells were incubated without test sample and with or without DMSO. The little percentage of DMSO present in the wells (maximal 0.1%) does not affect the experiment. After incubation of the cells at 37°C for 24 h, the viable cells yield was determined by a colorimetric method.

In brief, after the end of the incubation period, media were aspirated and the crystal violet solution (1%) was added to each well for at least 30 minutes. The stain was removed, and the plates were rinsed using tap water until all excess stain is removed. Glacial acetic acid (30%) was then added to all wells and mixed thoroughly, and then the absorbance of the plates were measured after gently shaken on Microplate reader (TECAN, Inc.), using a test wavelength of 490 nm. All results were corrected for background absorbance detected in wells without added stain. Treated samples were compared with the cell control in the absence of the tested compounds. All experiments were carried out in triplicate. The cell cytotoxic effect of each tested compound was calculated**.** The optical density was measured with the micro plate reader (Sun Rise, TECAN, Inc., USA) to determine the number of viable cells and the percentage of viability was calculated as [(OD_t_ / OD_c_)] x100% , where OD_t_ is the mean optical density of wells treated with the tested sample and OD_c_ is the mean optical density of untreated cells. The relation between surviving cells and drug concentration is plotted to get the survival curve of each tumor cell line after treatment with the specified compound.The 50% inhibitory concentration (IC_50_), the concentration required to cause toxic effects in 50% of intact cells, was estimated from graphic plots of the dose response curve for each conc. using Graph pad Prism software (San Diego, CA. USA)[4, 5].

### **Method S3 Antioxidant acivity**

Antioxidant Assay: The antioxidant activity of extract was determined at the Regional Center for Mycology and Biotechnology (RCMB) at Al- Azhar University *by* the DPPH free radical scavenging assay in triplicate and average values were considered.

DPPH Radical Scavenging Activity: Freshly prepared (0.004%w/v) methanol solution of 2,2-diphenyl-1-picrylhydrazyl (DPPH) radical was prepared and stored at 10 °C in the dark. The methanol solution of the free ligands and their complexes were prepared. A 40 μL aliquot of the methanol solution was added to 3 mL of DPPH solution. Absorbance measurements were recorded immediately with a UV-visible spectrophotometer (Milton Roy, Spectronic 1201). The decrease in absorbance at 515 nm was determined continuously, with data being recorded at 1 min intervals until the absorbance stabilized (16 min). The absorbance of the DPPH radical without antioxidant (control) and the reference compound ascorbic acid were also measured. All the determinations were performed in three replicates and averaged. The percentage inhibition (PI) of the DPPH radical was calculated according to the formula:

PI = [(*A*_C_- *A*_T_)/ *A*_C_] x 100 (1)

where *A*_C_ is the absorbance of the control at zero time and *A*_T_ is absorbance of the sample and DPPH at t = 16 min[6].

The 50% inhibitory concentration (IC_50_), the concentration required to inhibit DPPH radical by 50% was estimated from graphic plots of the dose response curve.

## **S.3. Figures, Schemes and Tables**

**Figure S 1: ^1^H-NMR for HL^1^, HL^2^, [Zn(L^1^)_2_].2H_2_O and [Zn(L^2^)_2_].2H_2_O in d_6_-DMSO**

**Figure S 2: ^13^C-NMR for organic ligands HL^1^, and HL^2^**

**
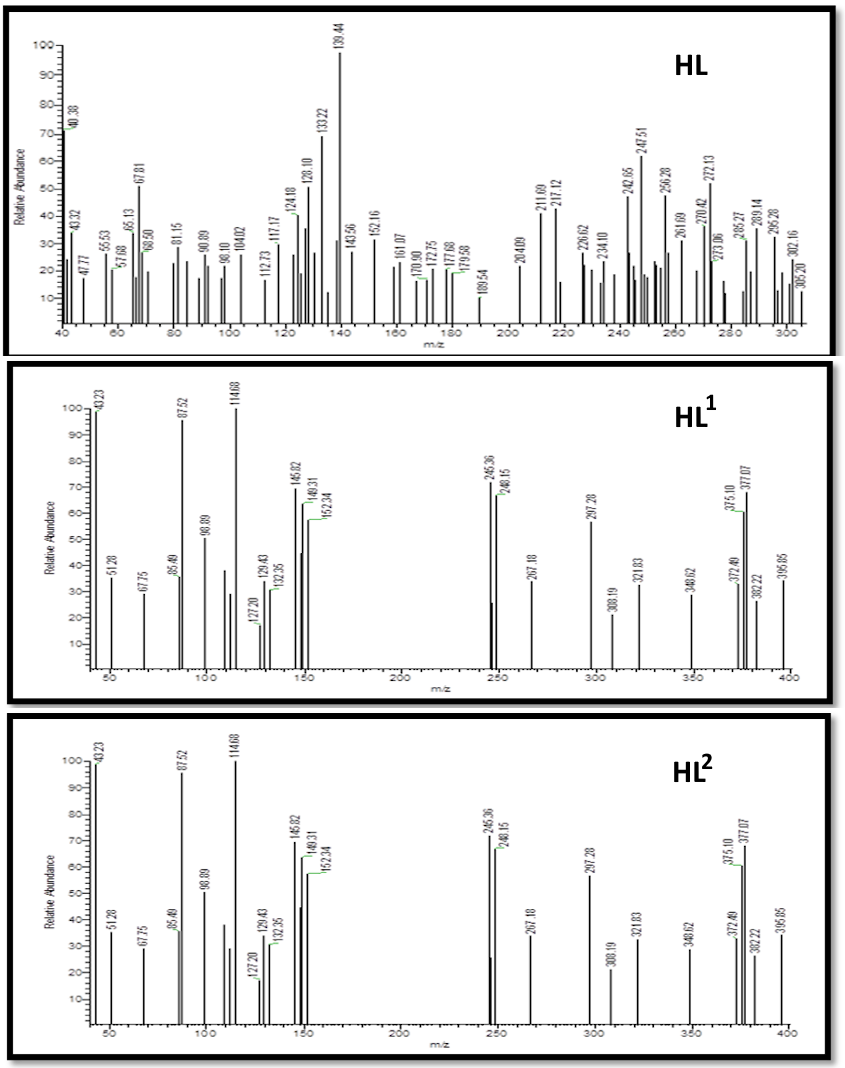
**

**Figure S 3: Mass spectra of HL, HL^1^, and HL^2^**

**
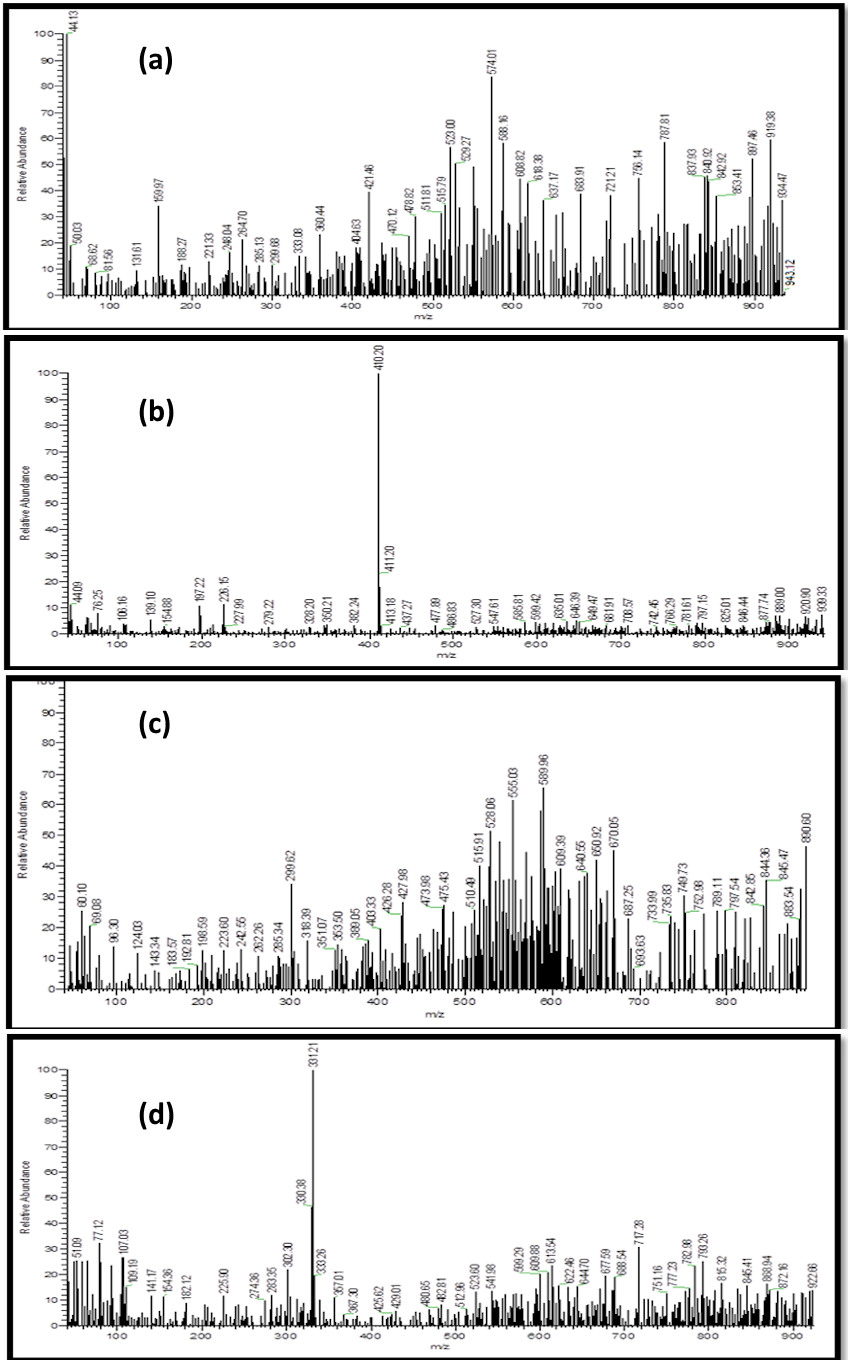
**

**Figure S 4: Mass Spectra of (a) [Cu(L^1^)₂].5H₂O, (b) [Cu(L^2^)₂].3H₂O, (c)[Zn(L^1^)₂].2H₂O, and [Zn(L^2^)₂].2H₂O.**

**Scheme S 1:****The possible fragmentation pathways of HL**

**Scheme S 2:** **The possible fragmentation pathways of HL^1^**

**Scheme S 3****: The possible fragmentation pathways of HL^2^**

**Scheme S 4:****The possible fragmentation pathways of [Cu(L^1^)₂].5H₂O**

**Scheme S 5:** **The possible fragmentation pathways of [Cu(L^2^)₂].3H₂O**

**Scheme S 6:** **The possible fragmentation pathways of [Zn(L^1^)₂].2H₂O**

**Scheme S 7:** **The possible fragmentation pathways of [Zn(L^2^)₂].2H₂O**

**
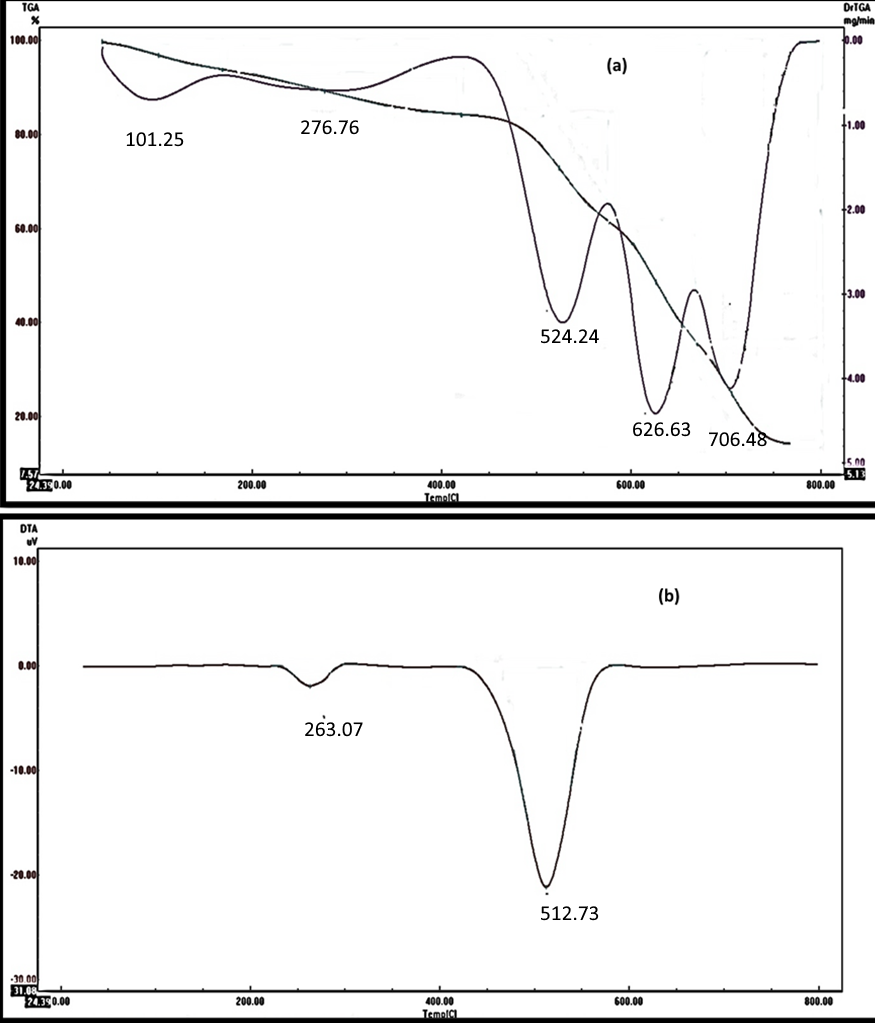
**

**Figure S 5****: Thermal analysis of HL: (a) [TGA DTG] and (b) [DTA]**

**
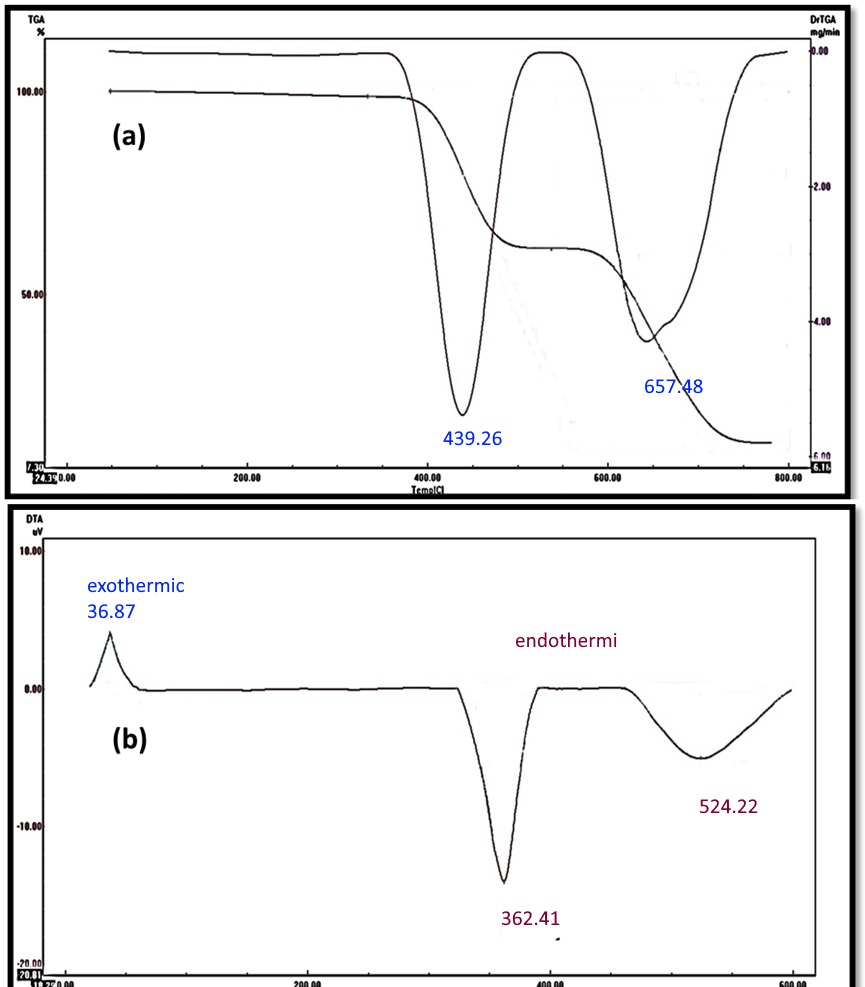
**

**Figure S 6:** **Thermal analysis of HL^1^, (a) [TGA DTG] and (b) [DTA]**

**
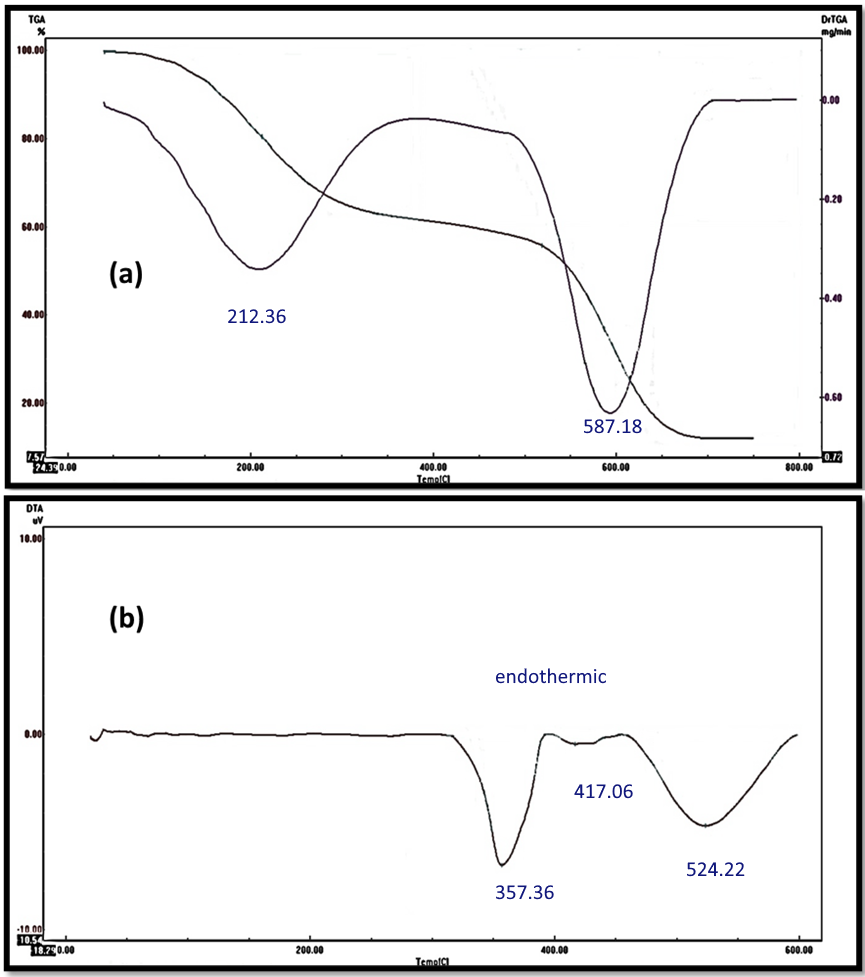
**

**Figure S 7: Thermal analysis of HL^2^: (a) [TGA DTG] and (b) [DTA]**

**
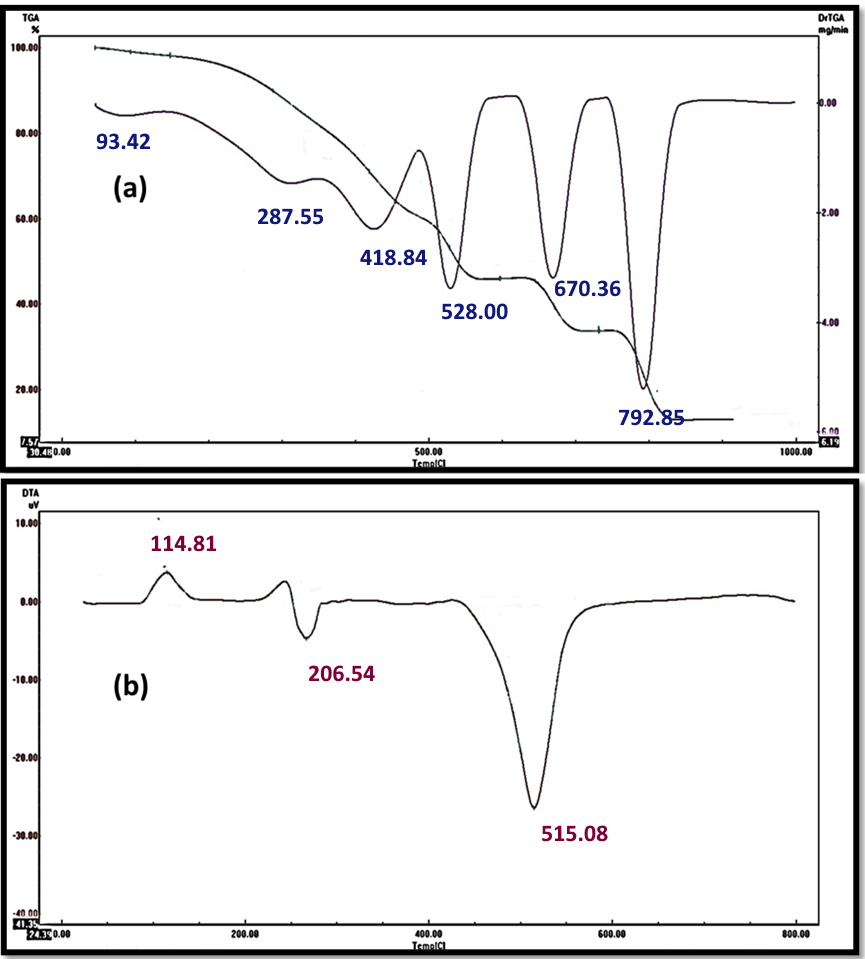
**

**Figure S 8:** **Thermal analysis of [Cu(L^1^)₂].5H₂O, (a) [TGA DTG] and (b) [DTA]**

**
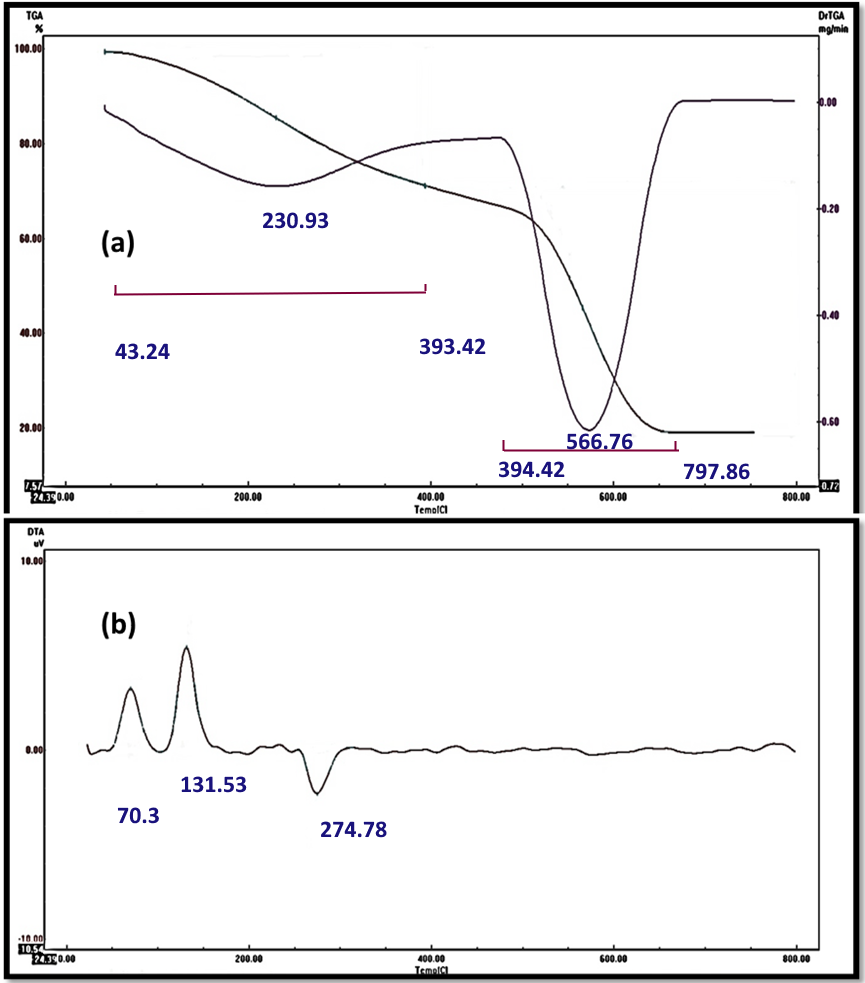
**

**Figure S 9:** **Thermal analysis of [Cu(L^2^)₂].3H₂O, (a) [TGA DTG] and (b) [DTA]**

**
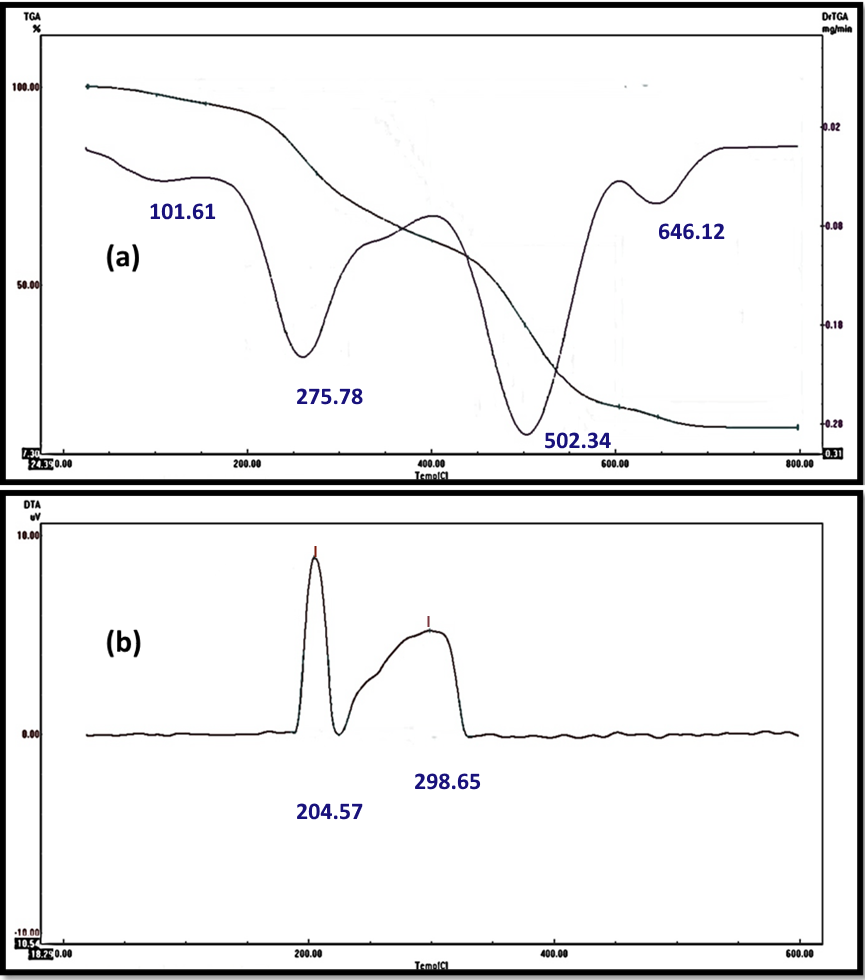
**

**Figure S 10:** **Thermal analysis of [Zn(L^1^)₂].2H₂O, (a) [TGA DTG] and (b) [DTA]**

**
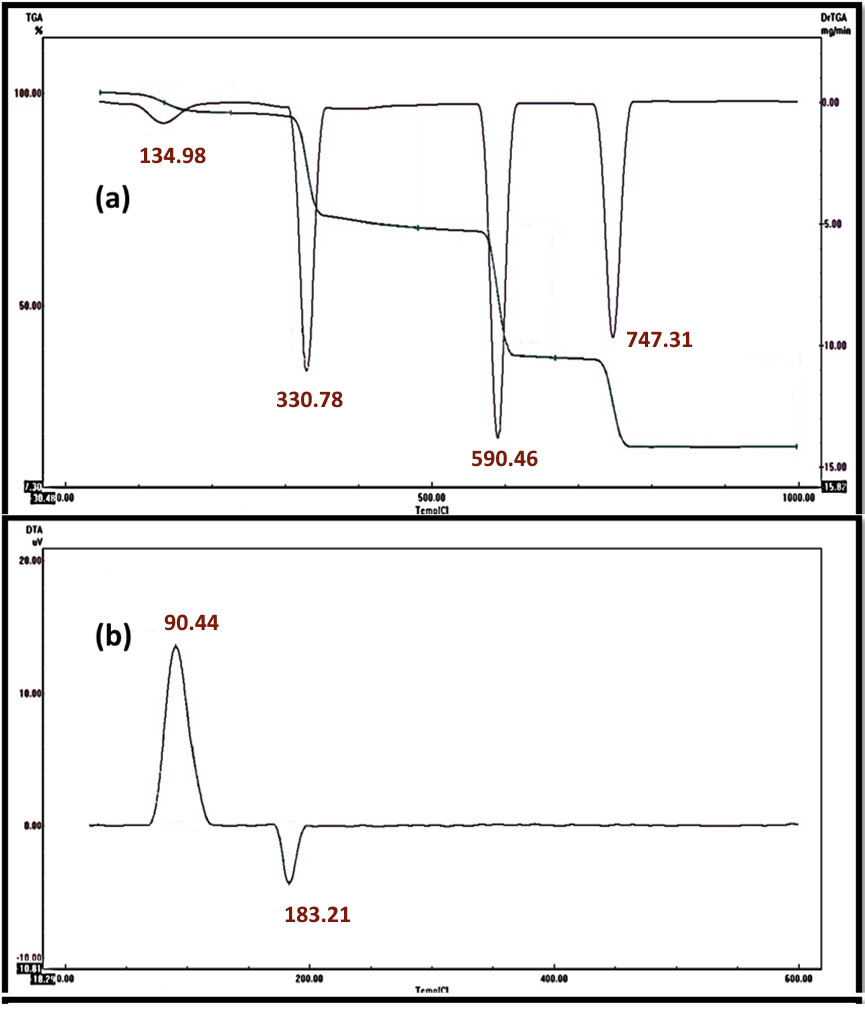
**

**Figure S 11:** **Thermal analysis of [Zn(L^2^)₂].2H₂O, (a) [TGA DTG] and (b) [DTA]**

**Scheme S 8:**  **The proposed thermal degradation of HL**

**Scheme S 9****: The proposed thermal degradation of HL^1^**

**Scheme S 10:** **The proposed thermal degradation of HL^2^**

**Scheme S 11:**  **The proposed thermal degradation of [Cu(L^1^)₂].5H₂O**

**Scheme S 12:** **The proposed thermal degradation of [Cu(L^2^)₂].3H₂O**

**Scheme S 13:** **The proposed thermal degradation of [Zn(L^1^)₂].2H₂O**

**Scheme S 14:The proposed thermal degradation of [Zn(L^2^)₂].2H₂O**

**Table S.1: Thermal analysis of the free ligands and their metal complexes.**

| Compound | Steps | TG  (^o^C) | DTG (^o^C) | Mass loss (%)  Found (Calc.) | Fragment | DTA  (^o^C) |
| --- | --- | --- | --- | --- | --- | --- |
| HL^1^ | I | 42.02-168.26 | 101.25 | 5.824 (5.58) | NH_3_ | 263.07  (Endo) |
|  | II | 168.93-420.53 | 276.76 | 9.576 (9.50) | CHO |  |
|  | III | 421.22-576.97 | 524.24 | 22.850 (22.28) | O_2_S + H_4_ | 512.73 (Endo) |
|  | IV | 577.28-670.03 | 626.63 | 25.751 (25.90) | C_5_H_3_O |  |
|  | V | 670.78-799.37 | 706.48 | 21.522 (20.98) | C_3_N_2_ |  |
|  |  | Residue |  | 14.229 (15.73) | C_4_ | --- |
| HL^2^ | I | 333.82-537.05 | 439.26 | 37.11 (37.12) | C_7_H_5_N_3_O | 36.87(Exo)  362.41 (Endo)  524.22 (Endo) |
|  | II | 537.80-799.75 | 657.48 | 48.215 (49.73) | C_6_H_6_NO_2_ + C_2_HO |  |
|  |  | Residue |  | 14.326 (13.13) | C_4_H_4_ |  |
| HL^3^ | I | 40.67-380.34 | 212.36 | 38.169 (38.35) | C_6_H_6_NO_2_S + H_2_ | 357.35 (Endo) |
|  | II | 380.34-797.29 | 587.18 | 49.898 (49.51) | C_9_H_6_N_3_OS | 417.06 (Endo)  524.22 (Endo) |
|  |  | Residue |  | 11.933(12.13) | C_4_H_2_ |  |
| [Cu(L^1^)₂].5H₂O | I | 45.76-147.66 | 93.42 | 1.862 (1.90) | H_2_O | 114.81 (Exo) |
|  | II | 148.60-351.19 | 287.55 | 16.345 (16.33) | H_2_NO_2_S + H_8_O_4_ | 206.54 (Endo) |
|  | III | 351.88-487.49 | 418.84 | 21.336 (21.31) | C_6_H_6_N_3_O_2_S + O + H |  |
|  | IV | 488.16-596.82 | 528.00 | 14.518 (14.42) | C_7_H_4_N_2_ + O + 4H | 515.08 (Endo) |
|  | V | 597.47-731.49 | 670.36 | 12.234 (12.40) | C_7_H_3_NO |  |
|  | VI | 732.45-997.62 | 792.85 | 20.693 (21.10) | C_10_H_5_N + C_5_ |  |
|  |  | Residue |  | 13.012 (12.50) | CuO + C_3_H_3_ |  |
| [Cu(L^2^)₂].3H₂O | I | 43.24-393.43 | 230.93 | 28.353(28.33) | C_6_H_6_NO_3_O_2_S +N_2_ + H_6_O_3_ | 70.3 (Exo)  131.53 (Exo)  274.78 (Endo) |
|  | II | 394.42-797.86 | 566.76 | 52.126(52.61) | C_6_H_6_NO_2_S + C_13_H_9_NOS + C_6_H_4_O + N +H_5_ |  |
|  |  | Residue |  | 19.521(19.04) | CuS + C_7_ |  |
| [Zn(L^1^)₂].2H₂O | I | 27.19-153.70 | 101.61 | 4.229(4.06) | H_4_O_2_ |  |
|  | II | 154.73-400.68 | 275.78 | 34.679(35.05) | C_12_H_12_N_2_O_4_S_2_ | 204.57 (Exo)  298.65 (Exo) |
|  | III | 400.68-603.18 | 502.34 | 42.074(42.03) | C_6_H_4_O+C_14_H_10_N_4_O_2_+C+H_4_ |  |
|  | IV | 604.15-798.70 | 646.12 | 5.254(4.94) | N_2_+O |  |
|  |  | Residue |  | 13.667(13.93) | Zn+C_5_ |  |
| [Zn(L^2^)₂].2H₂O | I | 47.47-224.75 | 134.98 | 4.759(4.12) | H_2_+H_4_O_2_ | 90.44 (Exo)  183.21 (Endo) |
|  | II | 225.72-480.81 | 330.78 | 27.069(27.11) | C_6_H_6_N_2_O_2_S+H_2_NO_2_S |  |
|  | III | 481.55-668.03 | 590.46 | 30.622(30.81) | C_13_H_8_N_2_O+C_6_H_4_ |  |
|  | IV | 668.66-997.51 | 747.31 | 21.006(21.04) | C_7_H_4_N_2_O+N+S+C+H_4_ |  |
|  |  | Residue |  | 16.544(16.90) | ZnS+C_5_ |  |

# **References**

[1] J. Bassett, J. Bassett, R. Denney, G. Jeffery, J. Mentham, Vogel's Textbook of Quantitative Inorganic Analysis: Including Elementry Instrumental Analysis, Longman ELBS1985.

[2] J. Hudzicki, Kirby-Bauer disk diffusion susceptibility test protocol, (2009).

[3] A. Bauer, W. Kirby, J.C. Sherris, M. Turck, Antibiotic susceptibility testing by a standardized single disk method, American journal of clinical pathology 45(4_ts) (1966) 493.

[4] T. Mosmann, Rapid colorimetric assay for cellular growth and survival: application to proliferation and cytotoxicity assays, Journal of immunological methods 65(1-2) (1983) 55.

[5] S.M. Gomha, S.M. Riyadh, E.A. Mahmmoud, M.M. Elaasser, Synthesis and anticancer activities of thiazoles, 1, 3-thiazines, and thiazolidine using chitosan-grafted-poly (vinylpyridine) as basic catalyst, Heterocycles 91(6) (2015) 1227.

[6] G.C. Yen, P.D. Duh, Scavenging effect of methanolic extracts of peanut hulls on free-radical and active-oxygen species, Journal of agricultural and food chemistry 42(3) (1994) 629.

1. Corresponding author: e-mail: [elbadawy_hm@yahoo.com](mailto:elbadawy_hm@yahoo.com); [hemmatabdelfattah@alexu.edu.eg](mailto:hemmatabdelfattah@alexu.edu.eg) [↑](#footnote-ref-1)
